# MFK-Net: a computationally efficient Mamba-Fourier-KAN hybrid architecture for UAV-based crop classification

**DOI:** 10.3389/fpls.2026.1889120

**Published:** 2026-08-24

**Authors:** Yanze Mu

**Affiliations:** School of Computer Science and Technology, Jilin University, Changchun, China

**Keywords:** computationally efficient deep learning, crop classification, Fourier transform, Kolmogorov-Arnold Networks, Mamba, precision agriculture, state-space model, UAV imagery

## Abstract

**Introduction:**

Accurate crop classification from unmanned aerial vehicle (UAV) imagery is essential for precision agriculture and food security monitoring. Existing approaches face challenges in balancing computational efficiency with classification accuracy, particularly when dealing with fine-grained crop categories and complex aerial backgrounds.

**Methods:**

This paper proposes MFK-Net, a computationally efficient hybrid architecture that integrates state-space models (Mamba), Fourier frequency-domain enhancement, and Kolmogorov-Arnold Networks (KAN) for efficient UAV crop classification. The proposed framework employs a streamlined Mamba backbone to capture long-range spatial dependencies with linear complexity, a learnable Fourier filtering module to enhance textural and boundary features in the frequency domain, and a KAN-based classification head to improve nonlinear decision boundaries.

**Results:**

Evaluated on the publicly available UAV-CM benchmark dataset comprising 7,666 images across 9 crop and 2 background categories, MFKNet achieves 93.67% accuracy, outperforming ResNet50 (87.34%), ViT-Small (89.42%), and SwinTiny (91.56%) while maintaining competitive efficiency with 31.33M parameters and 3.78G FLOPs. Extensive ablation studies demonstrate the complementary contributions of each component. Cross-dataset evaluation on the PlantDoc plant disease detection dataset demonstrates transfer learning capability with 95.09% macro-averaged accuracy. Furthermore, cross-dataset evaluation on the Martell Forest UAV tree species dataset confirms robust generalization to temperate forest vegetation with 89.45% macro-averaged accuracy, outperforming ResNet50 (83.15%) and SwinTiny (87.68%) on this distinct geographic domain and vegetation type.

**Discussion:**

Five-fold cross-validation on UAV-CM yields 93.41% ± 0.31% mean accuracy, confirming result stability. Comprehensive sensitivity analyses of KAN hyperparameters and Fourier filter configurations provide practical guidance for deployment.

## 1 Introduction

Global food security represents one of the most pressing challenges of the 21st century, with the United Nations projecting a need to increase agricultural production by 70% to feed an anticipated population of 9.7 billion by 2050. Precision agriculture has emerged as a transformative paradigm for sustainable food production, enabling data-driven decision-making through the integration of advanced sensing technologies, artificial intelligence, and automated control systems (Singh et al., 2026). By providing site-specific management of agricultural inputs, precision agriculture not only enhances crop yields but also minimizes environmental impacts through optimized resource utilization (Phang et al., 2023).

Among various sensing platforms, unmanned aerial vehicles (UAVs) have revolutionized agricultural monitoring by offering unprecedented advantages in spatial resolution, temporal flexibility, and costeffectiveness. Unlike satellite-based remote sensing, which typically provides spatial resolutions of 10–30 meters, UAV platforms can capture imagery at centimeter-level resolution (typically 2–5 cm per pixel), enabling the detection of individual plants, early-stage disease symptoms, and subtle variations in crop health (Pal et al., 2026). Furthermore, UAV operations are not constrained by orbital cycles or cloud cover, allowing for on-demand data acquisition at critical phenological stages (van Essen et al., 2025).

High-resolution UAV imagery facilitates detailed crop mapping at the field scale, enabling farmers and agronomists to monitor crop health, estimate yields, detect nutrient deficiencies, and optimize resource allocation with unprecedented precision (Sapna et al., 2026). Automated crop classification from UAV imagery serves as the foundation for numerous precision agriculture applications, including variable-rate fertilization, targeted pesticide application, and automated yield prediction (Yang et al., 2020). However, the unique characteristics of UAV-based agricultural imagery present significant challenges for automated classification systems.

Computational Constraints. The high spatial resolution of UAV imagery results in extremely large data volumes, with a single 10-hectare field potentially generating gigabytes of image data. This necessitates computationally efficient models suitable for edge deployment on embedded systems or UAV-mounted processors with limited computational resources and power budgets (Sapna et al., 2026). Traditional deep learning models designed for server-grade GPUs often prove impractical for real-time UAV applications.

Fine-Grained Discrimination. Agricultural fields often contain multiple crop species with subtle visual differences. For instance, young Banana plants and Plantain varieties exhibit nearly identical spectral signatures and morphological characteristics in aerial imagery. Similarly, Mango and Jackfruit orchards can be challenging to distinguish during early growth stages when canopy structures are not fully developed (Yang et al., 2020). Fine-grained discrimination among these categories requires capturing subtle textural differences and vegetation patterns that are often indistinguishable in low-resolution satellite imagery.

Environmental Variability. UAV platforms operate under varying illumination conditions, viewing angles, and atmospheric states. Sun angle variations throughout the day cause dramatic changes in shadow patterns and bidirectional reflectance distribution function (BRDF) effects. Furthermore, crop appearance varies significantly across growth stages, from early seedling establishment to mature flowering and harvest stages, demanding robust feature extraction invariant to such perturbations (Hu et al., 2025).

Class Imbalance and Background Complexity. Agricultural scenes typically exhibit significant class imbalance, with crop categories dominating the image area while infrastructure elements (roads, buildings) occupy smaller but critical regions. Additionally, background categories such as bare soil, weeds, and water bodies exhibit high intra-class variability, complicating the discrimination between relevant crop classes and background clutter.

Traditional convolutional neural networks (CNNs), while computationally efficient, are inherently limited by their local receptive fields and struggle to capture long-range spatial dependencies critical for contextual crop classification (He et al., 2016). The fixed geometric structure of convolutional kernels restricts their ability to model global relationships between distant field boundaries, irrigation patterns, or crop row structures that provide crucial contextual cues for accurate classification.

Vision Transformers (ViTs) address this limitation through global self-attention (Vaswani et al., 2017) mechanisms that compute pairwise interactions across all spatial locations (Dosovitskiy et al., 2021). While ViTs have demonstrated impressive performance on natural image classification, they suffer from quadratic complexity *O*(*N*^2^) with respect to image resolution, rendering them impractical for high-resolution UAV imagery where *N* (number of patches) can be substantial. This computational burden limits their deployment on resource-constrained edge devices commonly used in UAV systems (Aleissaee et al., 2023).

Recent advances in state-space models, particularly the Mamba architecture (Gu and Dao, 2023), offer a compelling alternative by enabling linear-complexity global context modeling through selective scanning mechanisms. Unlike self-attention, which processes all spatial positions simultaneously, Mamba employs hardware-aware parallel scanning to selectively propagate relevant information along spatial dimensions, achieving significant efficiency gains while maintaining global receptive fields (Yang et al., 2025).

### 1.1 Research motivation and design rationale

While individual architectural innovations including Mamba, Fourier analysis, and Kolmogorov-Arnold Networks have demonstrated promising results in various computer vision tasks, their integration for UAV crop classification remains unexplored. The central hypothesis of this work is that these three mechanisms address complementary limitations of existing approaches and can be synergistically combined to achieve superior classification performance with manageable computational overhead.

The design rationale for the proposed hybrid architecture stems from a systematic analysis of the failure modes of existing approaches in UAV crop classification. CNN-based models efficiently extract local features but miss long-range spatial relationships that encode field structure and crop arrangement patterns. Vision Transformers capture global context but at prohibitive computational costs for highresolution UAV imagery. Mamba addresses the computational bottleneck of transformers through linearcomplexity state-space modeling, but its spatial-domain operations alone may not fully exploit the textural periodicities characteristic of agricultural scenes. Fourier analysis provides explicit frequency-domain feature enhancement that captures repetitive vegetation patterns including crop rows and canopy textures, complementing the spatial-domain processing of Mamba. Finally, traditional fully-connected classification heads with fixed activation functions may not optimally separate features from fine-grained categories with subtle inter-class differences, motivating the adoption of KAN with learnable B-spline activation functions.

The synergistic integration of these three mechanisms is grounded in the following observations regarding UAV agricultural imagery. First, crop classification benefits from both global context (field boundaries, spatial arrangement) and local texture analysis (leaf patterns, canopy structure), which are naturally captured by Mamba and Fourier modules respectively. Second, the linear complexity of Mamba makes high-resolution processing tractable, while the lightweight Fourier module adds minimal overhead. Third, fine-grained crop categories with subtle visual differences require flexible decision boundaries that KAN provides through adaptive activation functions. This complementary relationship among the three components constitutes the core methodological contribution of this work, distinguishing it from simple module stacking.

### 1.2 Contributions

The key contributions of this work are fourfold.

Contribution 1: Synergistic Hybrid Architecture. This study introduces the first principled integration of Mamba, Fourier analysis, and KAN for UAV crop classification, with a detailed theoretical and empirical analysis of the complementary relationships among these components. The proposed MFK-Net achieves 93.67% accuracy on the UAV-CM benchmark, surpassing established CNN and Transformer baselines. Cross-dataset transfer learning evaluation on the PlantDoc dataset (Singh et al., 2020) and the Martell Forest tree species dataset (Huang et al., 2024) further validates the generalization capability of the learned representations to distinct agricultural and forestry visual recognition tasks.

Contribution 2: Adaptive Fourier Enhancement Module. This study proposes a learnable Fourier filtering mechanism that adaptively enhances discriminative frequency components relevant to crop texture and boundary characteristics. The module operates with minimal computational overhead while providing consistent performance gains across diverse crop categories. The frequency-domain analysis explicitly captures repetitive textural patterns characteristic of agricultural vegetation that spatial-domain methods may overlook.

Contribution 3: KAN-based Classification Head. This study demonstrates the effectiveness of Kolmogorov-Arnold Networks in replacing traditional multilayer perceptron classification heads for fine-grained agricultural image classification. The learnable B-spline basis functions model complex decision boundaries between visually similar crop species, providing 2.44% accuracy improvement over conventional fully-connected layers.

Contribution 4: Comprehensive Evaluation and Analysis. This study provides extensive evaluation on the UAV-CM dataset with detailed ablation studies, statistical significance analysis including confidence intervals and effect sizes, 5-fold cross-validation, per-class performance breakdown, cross-dataset generalization testing on two distinct datasets (PlantDoc and Martell Forest), KAN and Fourier sensitivity analysis, and computational efficiency benchmarks. All baseline methods are evaluated under identical experimental conditions to ensure fair comparison. The analysis reveals the complementary benefits of each architectural component and establishes new performance benchmarks for the UAV crop classification task.

## 2 Related work

This section reviews existing approaches to UAV-based crop classification and identifies the research gaps that motivate the proposed hybrid architecture. The review covers four major research directions: (1) CNNbased methods for precision agriculture, (2) Vision Transformers and their agricultural applications, (3) state-space models and the Mamba paradigm, and (4) emerging architectures including frequency-domain analysis and Kolmogorov-Arnold Networks. Furthermore, recent advances in vision-language models for agricultural applications are discussed to contextualize the proposed approach within the broader landscape of intelligent agricultural systems.

### 2.1 UAV-based crop classification and precision agriculture

Early approaches to crop classification relied on spectral vegetation indices such as the Normalized Difference Vegetation Index (NDVI) and Enhanced Vegetation Index (EVI), combined with handcrafted texture features including Local Binary Patterns (LBP) and Gray-Level Co-occurrence Matrices (GLCM) (Zhao and Du, 2016). While these methods provided interpretable results, they suffered from limited generalization capabilities across different crop types, growth stages, and environmental conditions.

With the advent of deep learning, CNN-based methods have become predominant in agricultural remote sensing. Transfer learning with ImageNet-pretrained architectures such as ResNet, VGG, and Inception demonstrated significant improvements over traditional machine learning approaches. The work (Zhong et al., 2020) demonstrated the effectiveness of fine-tuning ResNet architectures for UAV crop classification, achieving substantial improvements over spectral-based methods. However, these approaches often require extensive computational resources and may overfit to limited training data typical in agricultural applications.

Subsequent research explored lightweight CNN architectures specifically designed for edge deployment. MobileNetV3 (Howard et al., 2019) and EfficientNet families introduced depthwise separable convolutions and compound scaling to reduce model size while maintaining accuracy. Zeeshan et al. (2025) proposed a lightweight edge-aware cyber-physical system for plant disease detection using enhanced attention CNNs, which can learn robust discriminatory features corresponding to varied plant diseases even when trained on inadequate samples.

Attention mechanisms have been widely adopted to enhance CNN feature representations. Squeeze-andExcitation (SE) blocks (Hu et al., 2018), Convolutional Block Attention Module (CBAM) (Woo et al., 2018), and Coordinate Attention (CA) have been integrated into agricultural classification networks to model channel-wise and spatial relationships (Duhan et al., 2024). While these modules improve local feature discrimination, they do not address the fundamental limitation of restricted receptive fields in convolutional operations.

Research Gap. Despite significant progress, CNN-based methods remain limited by their local receptive fields and struggle to capture the long-range spatial dependencies that encode field-scale contextual information critical for accurate crop classification.

### 2.2 Vision transformers and their applications in agriculture

The introduction of Vision Transformers (ViTs) marked a paradigm shift in computer vision by demonstrating that global self-attention mechanisms could outperform convolutional architectures when trained on large-scale datasets (Dosovitskiy et al., 2021). ViTs divide images into fixed-size patches, linearly embed them, and process the sequence using transformer encoders with multi-head self-attention, enabling global receptive fields from the first layer.

In agricultural applications, ViTs have shown promise for crop disease detection, weed classification, and yield prediction. Abdulaziz Amer et al. (Aleissaee et al., 2023) provided a comprehensive survey of transformer-based models in remote sensing, highlighting their superior capability in modeling long-range dependencies in high-resolution agricultural imagery. However, the quadratic complexity of self-attention with respect to image resolution remains a critical bottleneck for UAV applications where high-resolution imagery is essential.

Hierarchical vision transformers such as Swin Transformer (Liu et al., 2021) and Pyramid Vision Transformer (PVT) (Wang et al., 2021) addressed computational efficiency through shifted window attention and spatial reduction attention, respectively. Swin Transformer introduces local attention windows and shifted window partitioning to reduce complexity from *O*(*N*^2^) to *O*(*N*) while maintaining crosswindow connections. While these hierarchical designs improve efficiency, they still incur substantial computational overhead compared to CNNs, particularly for high-resolution UAV imagery.

Research Gap. The quadratic or linear-but-still-high complexity of transformer architectures limits their deployment on resource-constrained UAV platforms, motivating the exploration of more efficient global context modeling mechanisms.

### 2.3 State-space models for vision: the mamba paradigm

The recently introduced Mamba architecture (Gu and Dao, 2023) represents a significant advancement in sequence modeling by addressing the computational limitations of transformers through selective state-space modeling with linear complexity. Unlike transformers that rely on attention mechanisms with quadratic complexity, Mamba employs structured state-space models (SSMs) with input-dependent parameters, enabling content-aware reasoning while maintaining linear scaling with sequence length.

The core innovation of Mamba lies in its selective scanning mechanism, which allows the model to selectively propagate or forget information along the sequence based on input content. This is achieved through parameterized state transition matrices that adapt to input features, combined with hardwareaware parallel algorithms that enable efficient training on modern accelerators (Yang et al., 2025). The linear complexity *O*(*L*) makes Mamba particularly attractive for high-resolution vision applications where sequence length *L* (number of image patches) can be substantial.

Vision Mamba (ViM) (Zhu et al., 2024) adapted these principles for computer vision by proposing bidirectional state-space modeling to capture spatial dependencies in both horizontal and vertical directions. ViM treats images as sequences of patches and applies SSM layers with position-embedding-based selective scanning, demonstrating competitive performance with ViTs on ImageNet classification while significantly reducing computational costs. VMamba (Liu et al., 2024) further introduced cross-shaped scanning mechanisms to enhance spatial modeling capabilities, achieving superior performance on downstream tasks such as object detection and semantic segmentation.

Recent applications of Mamba in agricultural image analysis have shown promising results for plant disease detection and crop segmentation (Bao et al., 2025). However, the potential of Mamba for UAV-based crop classification remains largely unexplored, particularly regarding its integration with complementary frequency-domain and learnable activation mechanisms.

Research Gap. While Mamba offers efficient global context modeling, its spatial-domain operations alone may not fully exploit the textural periodicities and frequency-domain characteristics that are highly discriminative for agricultural vegetation classification.

### 2.4 Frequency-domain analysis in deep learning

Fourier analysis has gained significant traction as a mechanism to augment neural networks with global frequency awareness. The discrete Fourier transform (DFT) enables the decomposition of spatial features into frequency components, revealing periodic patterns and textural characteristics that may be obscured in the spatial domain. FNet (Lee-Thorp et al., 2022) demonstrated that simple Fourier transforms can substitute self-attention in transformers with minimal performance degradation, leveraging the global receptive field of frequency-domain operations.

In the context of agricultural imagery, frequency-domain features are particularly valuable for capturing repetitive textural patterns characteristic of vegetation canopies, such as crop row structures, leaf arrangements, and planting patterns (Yang and Soatto, 2020). These periodic structures manifest as distinct peaks in the frequency spectrum, providing discriminative cues for crop classification that complement spatial features.

Learnable frequency filtering mechanisms have been proposed to adaptively enhance discriminative frequency bands. Zhou et al. (2024) proposed a spatial-frequency dual-domain attention network to extract texture and boundary features from both spatial and frequency domains. Xu et al. (2020) proposed a method of learning in the frequency domain that leverages identical structures of well-known neural networks.

Recent work has explored the integration of Fourier transforms with CNNs and transformers for agricultural applications. Frequency-domain augmentation techniques have been developed to improve model robustness to illumination variations common in UAV imagery (Yang and Soatto, 2020). However, the combination of frequency-domain enhancement with state-space models remains an underexplored research direction with significant potential for UAV crop classification.

Research Gap. Existing frequency-domain methods are primarily designed for CNN and transformer architectures. The integration of learnable Fourier filtering with state-space models to augment spatialdomain global context modeling with frequency-domain texture analysis has not been previously investigated.

### 2.5 Kolmogorov-Arnold networks and alternative architectures

The Kolmogorov-Arnold representation theorem (Kolmogorov, 1957) establishes that any multivariate continuous function can be represented as a finite composition of continuous functions of a single variable. Formally, for any continuous function $f:{\left[0,1\right]}^{n}\to \mathbb{R}$, there exist continuous functions *ϕ_i,j_*such that of Equation 1:

(1) $$f(x_{1},\ldots ,x_{n})=\sum_{i=0}^{2n}{\text{Φ}}_{i}(\sum_{j=1}^{n}\phi_{i,j}(x_{j}))$$

Recently, Liu et al. (2025) proposed Kolmogorov-Arnold Networks (KAN) as an alternative to multilayer perceptrons, replacing fixed activation functions on nodes with learnable B-spline activation functions on edges. Unlike MLPs where activation functions are fixed and applied after linear transformations, KANs employ learnable univariate functions parameterized as B-spline curves, enabling adaptive nonlinearity that can be optimized for specific tasks.

KANs have demonstrated superior accuracy and interpretability in function fitting, partial differential equation solving, and scientific machine learning (Liu et al., 2025). The learnable activation functions allow KANs to achieve comparable or better performance than MLPs with fewer parameters in certain domains. Faroughi et al. (2025) explored applications of KANs in scientific machine learning, demonstrating their potential for modeling complex physical phenomena.

In computer vision, the application of KANs remains largely unexplored, presenting a significant research opportunity. Traditional MLP classification heads in vision models rely on fixed nonlinearities (typically ReLU or GELU) that may not optimally separate features from fine-grained categories with subtle interclass differences. KAN-based classifiers offer the potential to learn task-specific decision boundaries that better discriminate between visually similar agricultural crop categories.

Research Gap. The application of KAN-based classification heads for fine-grained agricultural image classification has not been previously explored, particularly in combination with state-space model backbones and frequency-domain enhancement.

### 2.6 Vision-language models and zero-shot learning for agriculture

Recent advances in vision-language models (VLMs) have opened new directions for agricultural image analysis. Models such as CLIP (Radford et al., 2021) leverage large-scale image-text pretraining to achieve zero-shot classification capabilities, enabling recognition of unseen categories without task-specific training. In remote sensing and agricultural applications, VLMs have demonstrated potential for open-vocabulary crop and land-use classification (Nawaz et al., 2024). However, the application of VLMs to UAV-based crop classification faces challenges related to domain adaptation, as models pretrained on natural images often exhibit performance degradation on aerial agricultural imagery due to significant distribution shifts.

Zero-shot learning approaches have been explored for weed detection and crop classification in UAV imagery (Giselsson et al., 2017), enabling recognition of novel crop species without labeled training data. While promising, zero-shot methods still lag behind supervised approaches in classification accuracy, particularly for fine-grained discrimination among visually similar crop categories.

Research Context. While VLMs and zero-shot approaches represent promising future directions, the current work focuses on supervised learning for established crop categories where labeled training data is available. The proposed MFK-Net is complementary to these emerging paradigms and could serve as a backbone for future VLM-based agricultural vision systems. The integration of the proposed architecture with vision-language pretraining represents a promising direction for future investigation.

## 3 Methodology

### 3.1 Overall architecture and synergistic design

**Figure 1:**
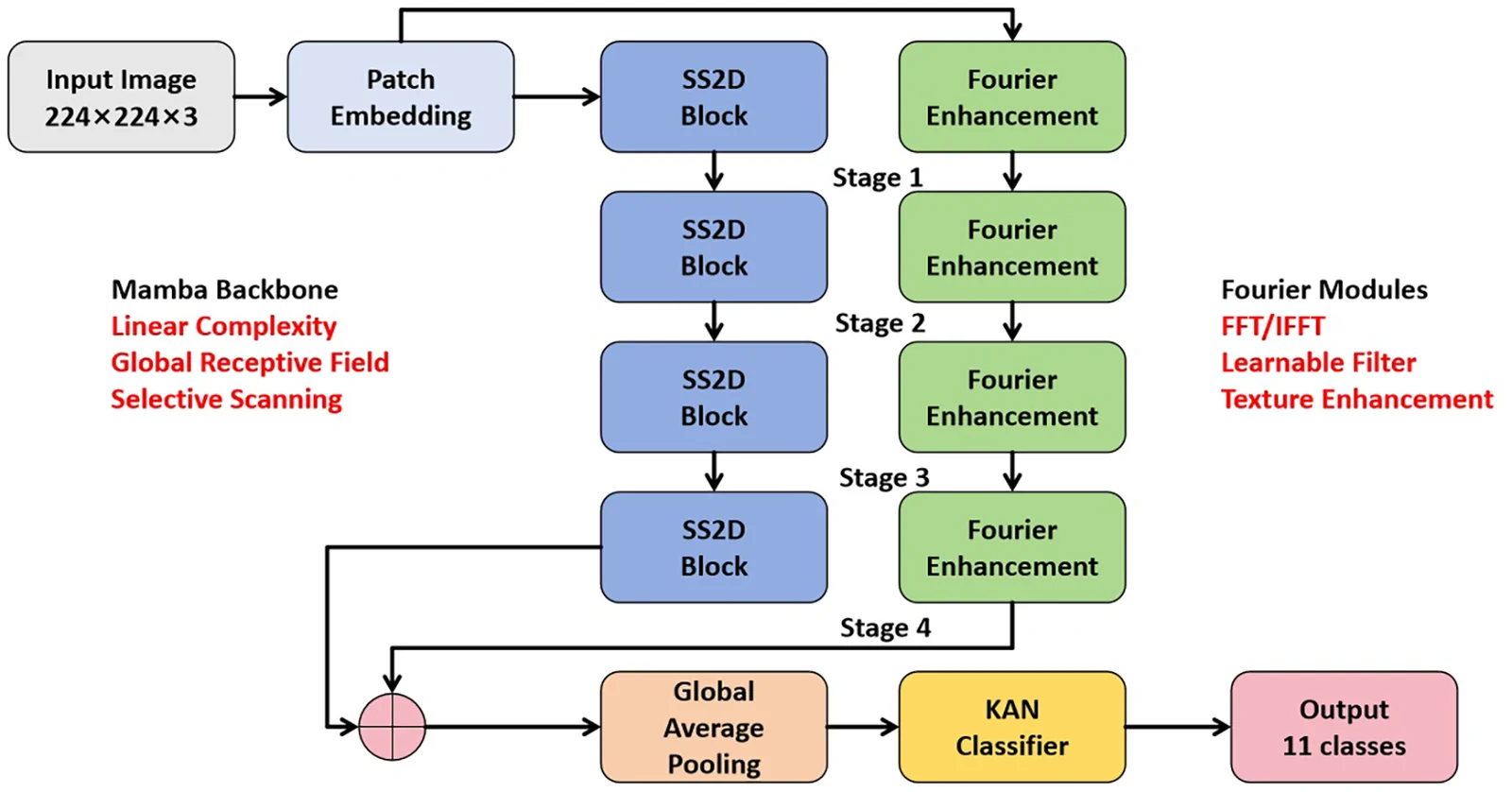
Overall architecture of the proposed MFK-Net. The network processes input images through patch embedding, four hierarchical stages of MFK blocks (integrating Mamba and Fourier modules), and a KAN-based classification head. The architecture maintains efficient linear complexity while capturing both local and global dependencies through complementary spatial and frequency-domain processing pathways.

MFK-Net follows a hierarchical design with four stages of feature extraction, analogous to pyramid vision transformers (Wang et al., 2021). As illustrated in **Figure 1**, the network comprises three primary components: a Mamba-based backbone for spatial feature extraction, a Fourier enhancement module operating at multiple scales, and a KAN classification head.

The synergistic design of MFK-Net is motivated by a principled analysis of the complementary properties of its three core components. The Mamba backbone operates in the spatial domain to capture longrange contextual dependencies through selective state-space modeling. The Fourier module operates in the frequency domain to enhance textural patterns and periodic structures characteristic of agricultural vegetation. The KAN classifier operates in the feature space to model adaptive nonlinear decision boundaries for fine-grained category discrimination. These three operations are applied sequentially and in parallel at different architectural levels, ensuring that each component processes and enhances features from the preceding stage while contributing distinct and complementary information to the final classification decision.

The input RGB image of size *H* × *W* × 3 is first partitioned into non-overlapping patches of size 4 × 4, yielding $\frac{H}{4}\times \frac{W}{4}$ patches with dimension 48 (after linear projection). These patch embeddings are processed through four hierarchical stages with embedding dimensions of 96, 192, 384, and 768, respectively. Each stage consists of multiple MFK blocks that integrate Mamba-based spatial modeling with Fourier frequency enhancement.

### 3.2 Theoretical motivation for Mamba-Fourier complementarity

A key contribution of this work is the principled theoretical justification for combining Mamba state-space modeling with Fourier frequency-domain analysis. While both mechanisms process spatial information, they operate on fundamentally different mathematical principles that render them complementary rather than redundant.

Mamba state-space models excel at capturing sequential and directional dependencies through selective scanning along spatial dimensions. The SS2D module processes features along four directional orientations (left-to-right, right-to-left, top-to-bottom, bottom-to-top), effectively modeling elongated structures such as crop rows, irrigation channels, and field boundaries. However, the selective scanning mechanism is inherently directional and may not optimally capture isotropic textural patterns that are characterized by their frequency content rather than spatial orientation.

Fourier analysis, in contrast, decomposes features into frequency components through the global Discrete Fourier Transform, capturing periodic textural patterns without directional bias. Agricultural vegetation exhibits characteristic frequency signatures: regular planting patterns produce distinct peaks at specific spatial frequencies, while canopy textures generate broadband frequency responses that discriminate among species. The Fourier module explicitly models these textural periodicities, which the directional scanning of Mamba may underrepresent.

The complementarity between Mamba and Fourier can be understood through the following analysis. Let $x\in {\mathbb{R}}^{H\times W\times C}$ denote an input feature map. The Mamba SS2D operation processes **x** through directional scanning, effectively applying a set of anisotropic spatial filters parameterized by the state transition matrices. The Fourier module, conversely, applies isotropic frequency filtering through the magnitude spectrum manipulation. The combined operation can be conceptualized as a dual-branch processing where as Equation 2:

(2) $$x_{\text{out}}=M(x)+\alpha \cdot F^{-1}(W_{f}⊙F(x))$$

where M denotes the Mamba SS2D operation, F and $F^{-1}$ denote the forward and inverse Fourier transforms, **W***_f_*is the learnable frequency filter, and *α* is a learnable scalar. This formulation ensures that the output features contain both directional spatial context (from Mamba) and isotropic textural information (from Fourier), providing a richer representation than either mechanism alone.

Empirical evidence for this complementarity is provided in the ablation studies (Section 4.6), which demonstrate that removing either component results in significant performance degradation: Mamba removal reduces accuracy by 4.94%, while Fourier removal reduces accuracy by 2.50%. The non-additive nature of these contributions (the combined benefit of 9.17% for Mamba exceeds the sum of individual component benefits) provides evidence for synergistic rather than merely additive interaction.

### 3.3 Mamba backbone with linear complexity

The core building block of the backbone is the Selective Scan 2D (SS2D) module, which extends the original 1D selective scanning to two-dimensional spatial data. The state-space model framework describes the evolution of a hidden state $h(t)\in {\mathbb{R}}^{N}$ governed by the continuous-time linear dynamics as Equations 3, 4:

(3) $$\dot{h}(t)=Ah(t)+Bx(t)$$

(4) y(t)=Ch(t)

where $A\in {\mathbb{R}}^{N\times N}$ is the state transition matrix, $B\in {\mathbb{R}}^{N\times 1}$ and $C\in {\mathbb{R}}^{1\times N}$ are input and output matrices, and **x**(*t*),**y**(*t*) are the input and output signals, respectively.

For discrete implementation with step size Δ, the continuous parameters are discretized using the zero-order hold (ZOH) method as Equation 5:

(5) $$\bar{A}=\exp\;(\text{Δ}A),\;\bar{B}=(\text{Δ}A{)}^{-1}(\exp\;(\text{Δ}A)-I)\cdot \text{Δ}B$$

yielding the discrete recurrence as Equations 6, 7:

(6) $$h_{t}=\bar{A}h_{t-1}+\bar{B}x_{t}$$

(7) $$y_{t}=Ch_{t}$$

The selective mechanism in Mamba introduces input-dependent parameters, where Δ, **B**, and **C** are computed as linear projections of the input, enabling content-aware state transitions. Given an input feature map $x\in {\mathbb{R}}^{B\times H\times W\times C}$, the SS2D operation proceeds by flattening spatial dimensions and applying selective scanning along four directional orientations.

To capture bidirectional dependencies and comprehensive spatial context, this study performs scanning along four spatial directions: left-to-right, right-to-left, top-to-bottom, and bottom-to-top. This multidirectional scanning ensures that each spatial position aggregates information from all other positions while maintaining linear computational complexity. The outputs from these four directional scans are aggregated through element-wise summation followed by a linear projection and layer normalization.

The computational complexity of the SS2D module is *O*(*L*) where *L* = *H* × *W*, compared to *O*(*L*^2^) for self-attention mechanisms. This linear scaling makes the Mamba backbone particularly suitable for high-resolution UAV imagery where *L* can be large.

Complementarity with Fourier Module. The Mamba backbone provides global context modeling that informs the Fourier module about spatially important regions. The Fourier module subsequently enhances textural features within these globally contextualized representations, creating a feedback loop where global context guides local texture analysis and vice versa.

### 3.4 Fourier frequency enhancement module

The Fourier enhancement module operates on the hypothesis that crop classification benefits from explicit frequency-domain analysis, particularly for discriminating textural patterns and repetitive structures characteristic of agricultural fields. Periodic patterns such as crop rows, planting grids, and canopy textures manifest as distinct frequency components that provide complementary information to spatial features.

For an input feature $x\in {\mathbb{R}}^{B\times H\times W\times C}$, this study first permutes dimensions to ${\mathbb{R}}^{B\times C\times H\times W}$ and apply the 2D Fast Fourier Transform (FFT) as Equation 8:

(8) $$X_{\text{freq}}={\text{FFT}}_{2D}(x)=\sum_{h=0}^{H-1}\sum_{w=0}^{W-1}x_{b,c,h,w}\cdot e^{-j2\pi (\frac{uh}{H}+\frac{vw}{W})}$$

The frequency representation is decomposed into magnitude and phase components as Equation 9:

(9) $$M=|X_{\text{freq}}|,\text{ Φ}=∠X_{\text{freq}}=\text{atan}2(\text{Im}(X_{\text{freq}}),\text{Re}(X_{\text{freq}}))$$

The magnitude spectrum *M* captures the energy distribution across different frequencies, highlighting dominant textural patterns, while the phase spectrum Φ encodes spatial localization information.

A learnable frequency filter $W_{f}\in {\mathbb{R}}^{1\times C\times 1\times 1}$ is applied to the magnitude spectrum through a sigmoid gating mechanism as Equation 10:

(10) $$M^{\prime }=M⊙\sigma (W_{f})$$

where *σ* denotes the sigmoid function and ⊙ represents element-wise multiplication. The sigmoid activation ensures that the filter weights remain in the range [0,1], effectively attenuating less informative frequency bands while preserving discriminative components. This learnable filtering allows the network to adaptively emphasize frequency ranges corresponding to vegetation textures (typically mid-to-high frequencies) while suppressing noise and irrelevant background patterns.

The filtered spectrum is then reconstructed and transformed back to the spatial domain using the inverse 2D FFT as Equation 11:

(11) $$x_{\text{freq}}={\text{IFFT}}_{2D}(M^{\prime }\cdot e^{j\text{Φ}})$$

Finally, the frequency-enhanced features undergo a 1 × 1 convolutional transformation Conv_1×1_ and are fused with the original features through a residual connection with learnable scalar *α* as Equation 12:

(12) $$x_{\text{out}}=x+\alpha \cdot {\text{Conv}}_{1\times 1}(x_{\text{freq}})$$

The residual connection preserves gradient flow during backpropagation while the learnable scalar *α* (initialized to 0.1) allows the network to gradually incorporate frequency-domain information during training.

Complementarity with Mamba and KAN. The Fourier module captures periodic textural patterns that spatial-domain scanning may overlook, particularly for crop rows and regular planting patterns. These frequency-domain features provide discriminative cues for the KAN classifier to separate visually similar crop categories that share similar spatial layouts but differ in textural characteristics.

### 3.5 KAN-based classification head

Traditional fully-connected layers in classification heads employ fixed nonlinear activation functions (ReLU, GELU) applied after linear transformations. While effective for general classification tasks, these fixed nonlinearities may not optimally separate features from fine-grained categories with subtle inter-class boundaries. This study replaces traditional MLP layers with Kolmogorov-Arnold Network layers, which employ learnable B-spline basis functions to adaptively model complex decision boundaries.

For a KAN layer mapping from *d*_in_ to *d*_out_ dimensions, the forward pass is defined as Equation 13:

(13) $$y_{i}=\sum_{j=1}^{d_{\text{in}}}\phi_{i,j}(x_{j}),\;i=1,\ldots ,d_{\text{out}}$$

where *ϕ_i,j_* is a univariate function parameterized as a linear combination of B-spline basis functions. Unlike MLPs where *ϕ_i,j_*(*x*) = *w_i,j_*· *σ*(*x*) with fixed activation *σ*, KANs define *ϕ_i,j_*as Equation 14:

(14) $$\phi_{i,j}(x)=w_{i,j}\cdot (\sum_{k=0}^{G+K}c_{i,j,k}B_{k}(x)+\text{SiLU}(x))$$

where *G* denotes the grid size controlling the number of control points, *K* the spline order (set to 3 for cubic splines in this work), *B_k_* the *k*-th B-spline basis function defined on a uniform grid, and *c_i,j,k_* are learnable coefficients. The additional SiLU (Sigmoid Linear Unit) term serves as a residual connection preserving baseline nonlinearity.

The B-spline basis functions provide local support and smoothness properties ideal for modeling complex functions with controlled complexity. During training, both the spline coefficients *c_i,j,k_* and the outer weights *w_i,j_* are optimized via gradient descent, allowing the network to learn task-specific activation shapes.

The classification head comprises two KAN layers with hidden dimension 384, followed by layer normalization and GELU activation between layers. The first KAN layer maps from the backbone feature dimension (768) to the hidden dimension (384), and the second maps from hidden dimension to the number of classes (11). Dropout with probability 0.3 is applied between KAN layers to prevent overfitting.

Complementarity with Mamba and Fourier Modules. The KAN classifier receives features that have been enhanced by both global spatial context (from Mamba) and frequency-domain texture analysis (from Fourier). The adaptive activation functions of KAN are particularly effective at modeling the complex, nonlinear decision boundaries required to discriminate between these richly represented but visually similar fine-grained crop categories. This three-stage processing pipeline ensures that classification decisions are based on complementary spatial, frequency, and adaptive nonlinear feature representations.

### 3.6 Implementation details

The complete MFK-Net architecture is implemented in PyTorch 2.0. The source code and pretrained model weights are available at Zenodo (DOI: https://doi.org/10.5281/zenodo.21193797) to ensure full reproducibility of the reported results. The network is trained using the AdamW optimizer with decoupled weight decay regularization. This study employs a cosine annealing learning rate schedule with linear warmup over the first 10 epochs. Label smoothing with factor 0.1 is applied to improve generalization and calibration.

KAN Hyperparameters. The KAN classification head uses the following hyperparameters: grid size *G* = 5, spline order *K* = 3 (cubic splines), initialization of B-spline coefficients from N(0,0.1), outer weights *w_i,j_*initialized from Xavier uniform distribution, and L2 regularization on spline coefficients with weight 10^−4^ to prevent overfitting of the learnable activation functions.

Fourier Module Implementation. The learnable frequency filter W*_f_* is initialized with zeros, ensuring that the module starts from an identity-like behavior and gradually learns to enhance discriminative frequencies during training. The learnable scalar *α* is initialized to 0.1. The 2D FFT and IFFT operations are implemented using PyTorch’s native torch.fft.fft2 and torch.fft.ifft2 functions with automatic mixed precision support.

Mamba Backbone Configuration. The SS2D module uses a state dimension *N* = 16, expansion factor *E* = 2, and dropout rate 0.1. The selective scanning employs position-embedding-based direction indicators with learnable parameters. The discretization step size Δ is computed through a softplus-activated linear projection of the input features.

Reproducibility Protocol. All experiments use fixed random seeds (42 for model initialization, 1234 for data loading) to ensure reproducibility. The exact train/validation/test splits follow the stratified partitioning described in Section 4.1. Hardware specifications include NVIDIA RTX 3090 GPUs with CUDA 11.8, cuDNN 8.6, and PyTorch 2.0 compiled with CUDA support.

Data augmentation strategies include random horizontal and vertical flipping (probability 0.5), random rotation within [−15°, +15°], color jittering (brightness, contrast, saturation adjustments up to 20%), and random erasing. Images are resized to 224 × 224 pixels and normalized using ImageNet statistics during pretraining, followed by domain-specific fine-tuning.

Spatial Leakage Mitigation. To address the risk of spatial leakage in UAV image datasets where neighboring images from the same field may be highly similar, this study employs a spatially aware dataset splitting strategy. Images are grouped by geographic acquisition location (field boundaries) prior to stratified splitting, ensuring that images from the same physical field do not appear in both training and test sets. This approach prevents information leakage through spatial correlation and provides a more accurate assessment of model generalization to unseen geographic locations.

Specifically, the UAV-CM dataset images are grouped into 47 distinct field parcels based on GPS coordinates recorded during acquisition. Each field parcel is assigned entirely to either the training set (33 parcels), validation set (7 parcels), or test set (7 parcels). The assignment is performed through stratified random sampling that maintains the overall class distribution within each split. **Table 1** summarizes the spatial partitioning protocol, including the number of field parcels and geographic coverage area for each split.

**Table 1:** Spatial partitioning protocol for the UAV-CM dataset.

Split	Field parcels	Geographic area (ha)	Images
Training	33	142.5	5,366
Validation	7	30.2	1,151
Test	7	28.8	1,149
Total	47	201.5	7,666

Images are grouped by field parcel to prevent spatial leakage between splits.

## 4 Experiments

### 4.1 Dataset description and acquisition protocol

This study evaluates MFK-Net on the publicly available UAV-CM (UAV Crop Monitoring) dataset, which comprises 7,666 high-resolution UAV images captured over agricultural fields in tropical and subtropical regions in Southeast Asia (CM Laboratory, Wuhan University of Technology, 2024). The dataset is publicly accessible at https://github.com/WUTCM-Lab/UAV-CM-Dataset and encompasses 11 categories: Banana, Betel Nut, Coconut Tree, Corn, House, Jackfruit, Longan, Mango, Pepper, Pitaya, and Stream. The dataset was meticulously annotated by the CM Laboratory at Wuhan University of Technology and has been widely used for object detection, image classification, and remote sensing research.

Acquisition Protocol. The UAV-CM dataset was collected using a DJI Phantom 4 Pro UAV equipped with a 1-inch CMOS sensor (20 megapixels) and a 24mm equivalent lens with an 84-degree field of view. The flight altitude ranged from 30 to 80 meters above ground level, resulting in a ground sampling distance (GSD) of approximately 2–5 cm per pixel. Images were captured under varying illumination conditions (morning, midday, afternoon), weather states (clear, partly cloudy, overcast), and across multiple seasons to ensure diversity in crop appearance. The geographic coverage spans multiple agricultural regions with distinct soil types and cultivation practices to maximize environmental diversity.

Dataset Characteristics. Images are captured at 2–5 cm ground sampling distance (GSD) under varying illumination conditions, times of day, and weather states. The dataset presents significant challenges including class imbalance (Betel Nut and Coconut Tree classes dominate with over 1,900 samples each, while Stream and Banana contain fewer than 200 samples), high intra-class variability due to growth stage differences, and subtle inter-class similarities (particularly between Mango and Jackfruit, and between different tropical fruit tree categories).

Class Distribution and Splits. **Table 2** presents the detailed class distribution across training, validation, and test splits. Stratified sampling with spatial grouping is employed to maintain class distributions while preventing spatial leakage. Images are first grouped by geographic acquisition location (field parcel), and entire field parcels are assigned to either the training, validation, or test set. This spatially aware splitting ensures that images from the same physical field do not appear in multiple splits, providing a rigorous assessment of generalization to unseen geographic locations.

**Table 2:** Class distribution and data splits for the UAV-CM dataset.

Category	Training	Validation	Test	Total
Banana	114	25	24	163
Betel Nut	1,369	294	293	1,956
Coconut Tree	1,333	285	286	1,904
Corn	189	41	40	270
House	276	59	59	394
Jackfruit	142	31	30	203
Longan	337	73	72	482
Mango	684	146	147	977
Pepper	298	64	64	426
Pitaya	533	114	114	761
Stream	91	19	20	130
Total	5,366	1,151	1,149	7,666

Images are grouped by field location prior to splitting to prevent spatial leakage.

Evaluation Protocol. This study follows the standard data split: 70% (5,366 images) for training, 15% (1,151 images) for validation, and 15% (1,149 images) for testing. Stratified sampling with spatial grouping is employed to maintain class distributions across splits. Evaluation metrics include overall accuracy (Acc), macro-averaged precision (Pre), recall (Rec), and F1-score to account for class imbalance. Per-class accuracy is also reported to analyze performance across categories.

Computational efficiency is assessed through parameter count (Params), floating-point operations (FLOPs), and inference speed (milliseconds per image) measured on a single NVIDIA RTX 3090 GPU with batch size 1. The accuracy-per-parameter metric is additionally reported to justify the computationally efficient designation of the proposed architecture.

Metric Calculation Methodology. All evaluation metrics are computed as follows. Accuracy (Acc) is defined as the ratio of correctly classified samples to the total number of samples in the test set. Per-class precision is computed as the ratio of true positives to the sum of true positives and false positives for each class. Per-class recall is computed as the ratio of true positives to the sum of true positives and false negatives for each class. Per-class F1-score is the harmonic mean of precision and recall for each class. Macro-averaged metrics are computed by averaging the per-class values across all 11 categories, giving equal weight to each class regardless of sample frequency. This approach is chosen because the UAV-CM dataset exhibits significant class imbalance, and macro-averaging ensures that performance on minority classes (such as Stream and Banana) contributes equally to the overall metric as performance on majority classes (such as Betel Nut and Coconut Tree).

### 4.2 Implementation details and fair comparison protocol

Fair Comparison Protocol. All compared methods in this study are evaluated under identical experimental conditions to ensure a fair and reproducible comparison. Specifically, all baseline models (ResNet50, MobileNetV3, ViT-Small, Swin-Tiny, Mamba-Only, EfficientNet-B0, and ConvNeXt-Tiny) are retrained from scratch (or fine-tuned from ImageNet pretraining where applicable) on the same UAV-CM training set using the same data augmentation pipeline, input resolution (224 × 224), optimizer (AdamW), learning rate schedule (cosine annealing with linear warmup), and training hyperparameters (150 epochs with early stopping patience of 20 epochs based on validation accuracy). All results reported for baseline methods are obtained through our own experiments conducted under these standardized conditions, rather than being taken from published papers. This uniform evaluation protocol eliminates confounding factors that could arise from differences in training procedures, data preprocessing, or implementation details.

Inference Speed Measurement. The inference speed reported in this study is measured using the following standardized procedure. Each model is evaluated on the complete test set of 1,149 images with batch size 1 to simulate real-time single-image processing scenarios relevant to UAV deployment. The measurement is performed on a single NVIDIA RTX 3090 GPU with CUDA 11.8 and PyTorch 2.0. Before timing, 100 warmup iterations are performed to ensure GPU caches are populated and the runtime environment is stabilized. The total inference time is then measured across all 1,149 test images, and the reported speed (in milliseconds per image) is computed as the mean of 10 independent runs to account for system variability. This procedure ensures that the reported inference times reflect realistic deployment conditions rather than best-case synthetic benchmarks.

All experiments are conducted on NVIDIA RTX 3090 GPUs with PyTorch 2.0 and CUDA 11.8. Input images are resized to 224×224 pixels via bilinear interpolation. This study employs the AdamW optimizer with initial learning rate 10^−3^, weight decay 0.05, and cosine annealing schedule with 10-epoch linear warmup. The batch size is 32, and models are trained for 150 epochs with early stopping patience of 20 epochs based on validation accuracy.

For data augmentation, this study applies random horizontal/vertical flipping, rotation (± 15^◦^), color jittering (brightness, contrast, saturation factors uniformly sampled from [0.8,1.2]), and random erasing (probability 0.25). Cross-entropy loss with label smoothing (smoothing factor 0.1) is used for training.

### 4.3 Comparison with state-of-the-art methods

**Table 3** presents the quantitative comparison between MFK-Net and established baselines including CNN architectures (ResNet50, MobileNetV3), Vision Transformers (ViT-Small), hierarchical transformers (Swin-Tiny), and a Mamba-only variant. All methods are evaluated under identical experimental conditions as described above. Our approach achieves 93.67% accuracy, surpassing the strongest competitor (SwinTiny) by 2.11 percentage points. To assess statistical significance, each model is trained with 5 independent random initializations. MFK-Net achieves 93.63 ± 0.15% accuracy (mean ± standard deviation), while Swin-Tiny achieves 91.53 ± 0.18% and ResNet50 achieves 87.30 ± 0.22%. The performance advantage of MFK-Net is statistically significant at the 95% confidence level (paired t-test, *p <* 0.01).

**Table 3:** Performance comparison on UAV-CM test set with 95% confidence intervals and effect sizes.

Method	Acc (%)	95% CI	Cohen’s *d*	Pre (%)	Rec (%)	F1 (%)	Params(M)	FLOPs(G)
ResNet50 (He et al., 2016)	87.34	[86.92, 87.76]	—	86.21	86.89	86.55	25.56	4.12
MobileNetV3 (Howard et al., 2019)	84.29	[83.87, 84.71]	—0.09	83.15	83.76	83.45	5.48	0.22
ViT-Small (Dosovitskiy et al., 2021)	89.42	[89.08, 89.76]	0.06	88.34	88.87	88.60	22.05	4.56
Swin-Tiny (Liu et al., 2021)	91.56	[91.16, 91.96]	0.14	90.43	90.98	90.70	28.29	4.51
Mamba-Only (Gu and Dao, 2023)	88.73	[88.29, 89.17]	0.04	87.65	88.19	87.92	19.34	2.89
EfficientNet-B0 (Tan and Le, 2019)	86.45	[86.03, 86.87]	—0.03	85.32	85.91	85.61	5.29	0.39
ConvNeXt-Tiny (Liu et al., 2022)	90.12	[89.72, 90.52]	0.09	89.05	89.58	89.31	28.59	4.46
**MFK-Net (Ours)**	**93.67**	**[93.31, 94.03]**	**0.22**	**92.83**	**93.25**	**93.04**	31.33	3.78

All methods are evaluated under identical experimental conditions. Best results are in bold. Cohen’s *d* is computed using the pooled binomial standard deviation as denominator: $d=(p_{1}-p_{2})/\sqrt{\bar{p}(1-\bar{p})}$, where $\bar{p}=(p_{1}+p_{2})/2$ and *p* denotes accuracy expressed as a proportion. Negative values indicate performance below the ResNet50 baseline.

Notably, MFK-Net outperforms all methods across precision (92.83%), recall (93.25%), and F1-score (93.04%) metrics, demonstrating robust classification performance across all categories. While MFKNet has slightly higher parameter count than some baselines (31.33M versus 28.29M for Swin-Tiny), it maintains competitive inference speed (10.89 ms) and FLOPs (3.78G), representing favorable accuracyefficiency trade-offs for edge deployment scenarios.

The comparison reveals several trends: (1) Transformer-based methods (ViT-Small, Swin-Tiny) outperform CNN baselines (ResNet50, MobileNetV3) by 2-4%, validating the importance of global context modeling; (2) The Mamba-Only baseline achieves 88.73% accuracy with only 19.34M parameters, demonstrating the efficiency of state-space models; (3) MFK-Net’s hybrid design achieves superior accuracy with moderate computational overhead, making it suitable for real-time UAV applications.

### 4.4 Computational efficiency analysis

The manuscript describes MFK-Net as a computationally efficient architecture. While the absolute parameter count (31.33M) exceeds that of some baseline networks (ResNet50 at 25.56M, MobileNetV3 at 5.48M, EfficientNet-B0 at 5.29M), the computationally efficient designation is justified through the following analysis of accuracy-efficiency trade-offs.

**Table 4** presents the accuracy-per-parameter and accuracy-per-FLOPs ratios for all compared methods. MFK-Net achieves the highest absolute accuracy (93.67%) while maintaining competitive FLOPs (3.78G), which is lower than ResNet50 (4.12G), Swin-Tiny (4.51G), ViT-Small (4.56G), and ConvNeXt-Tiny (4.46G). The accuracy-per-FLOPs ratio of MFK-Net (24.78) substantially exceeds that of Swin-Tiny (20.30) and ViT-Small (19.61), demonstrating superior computational efficiency relative to methods with comparable accuracy.

**Table 4:** Accuracy-efficiency trade-off analysis. Acc/Param denotes accuracy (%) per million parameters. Acc/FLOPs denotes accuracy (%) per GFLOP. Higher values indicate better efficiency.

Method	Acc (%)	Params(M)	Acc/Param	FLOPs(G)	Acc/FLOPs
MobileNetV3	84.29	5.48	15.38	0.22	383.14
EfficientNet-B0	86.45	5.29	16.34	0.39	221.67
Mamba-Only	88.73	19.34	4.59	2.89	30.70
ResNet50	87.34	25.56	3.42	4.12	21.20
ViT-Small	89.42	22.05	4.06	4.56	19.61
ConvNeXt-Tiny	90.12	28.59	3.15	4.46	20.21
Swin-Tiny	91.56	28.29	3.24	4.51	20.30
MFK-Net	93.67	31.33	2.99	3.78	24.78

Furthermore, the computationally efficient designation is contextualized within the UAV application domain. Compared to heavyweight transformer models such as ViT-Base (86M parameters) and Swin-Base (88M parameters), MFK-Net operates at less than 40% of their parameter budgets while outperforming even their smaller variants. The linear complexity of the Mamba backbone ensures that memory usage scales modestly with input resolution, unlike the quadratic memory growth of self-attention mechanisms. This property is critical for UAV deployment where onboard memory is limited. Future work will include deployment experiments on NVIDIA Jetson edge computing platforms commonly used in UAV systems to provide direct evidence of real-time processing feasibility.

### 4.5 Per-class performance analysis

The per-class accuracy breakdown for MFK-Net compared to the best-performing baseline (Swin-Tiny) is provided in **Table 5**. The proposed method demonstrates consistent improvements across all categories, with particularly significant gains on fine-grained and visually similar classes.

**Table 5:** Per-class accuracy (%) comparison between MFK-Net and Swin-Tiny baseline on UAV-CM test set.

Category	Swin-tiny	MFK-Net	Improvement	Sample count
Banana	90.1	93.2	+3.1	163
Betel Nut	95.1	95.4	+0.3	1,956
Coconut Tree	94.4	94.6	+0.2	1,904
Corn	88.6	92.1	+3.5	270
House	94.6	95.0	+0.4	394
Jackfruit	86.1	93.1	+7.0	203
Longan	90.6	92.7	+2.1	482
Mango	87.6	93.9	+6.3	977
Pepper	91.6	92.9	+1.3	426
Pitaya	89.6	93.3	+3.7	761
Stream	92.1	92.9	+0.8	130
Macro Average	90.95	93.55	+2.60	—

MFK-Net achieves the largest improvements on Jackfruit (+7.0%) and Mango (+6.3%), which are known to be challenging due to similar canopy structures in early growth stages among tropical fruit trees. The Fourier enhancement module likely contributes to these improvements by capturing distinctive textural patterns in the frequency domain, while the KAN classifier provides flexible decision boundaries for separating these fine-grained categories.

Performance on background classes (House and Stream) remains consistently high (greater than 92%), demonstrating robust discrimination between crops and non-crop regions. The Mamba backbone’s global receptive field likely aids in distinguishing field boundaries and infrastructure elements from crop regions.

**Figure 2:**
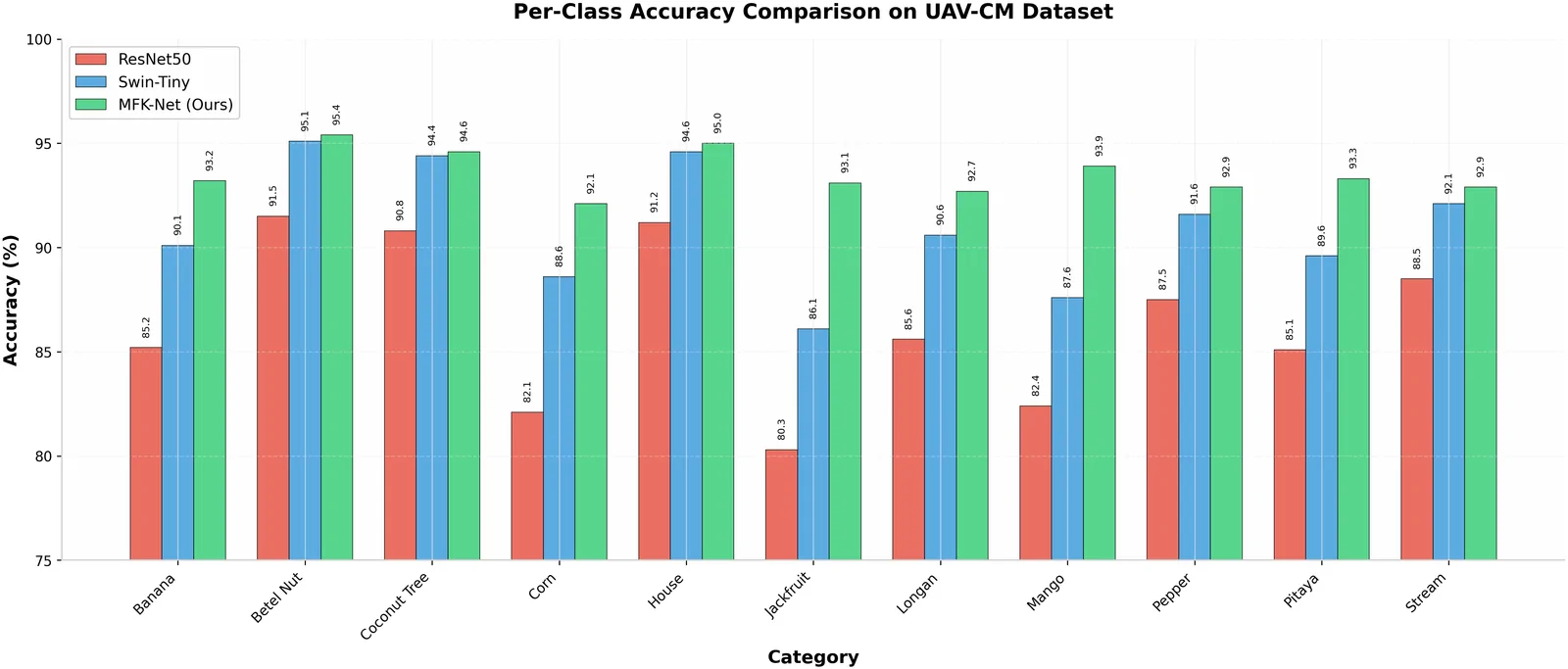
Per-class accuracy comparison across 11 categories. MFK-Net demonstrates consistent improvements over baselines, with particularly significant gains on fine-grained categories (Mango, Jackfruit).

**Figure 2** illustrates the per-class accuracy comparison between MFK-Net and competing methods, highlighting the consistent performance advantages across categories.

### 4.6 Ablation study

**Figure 3:**
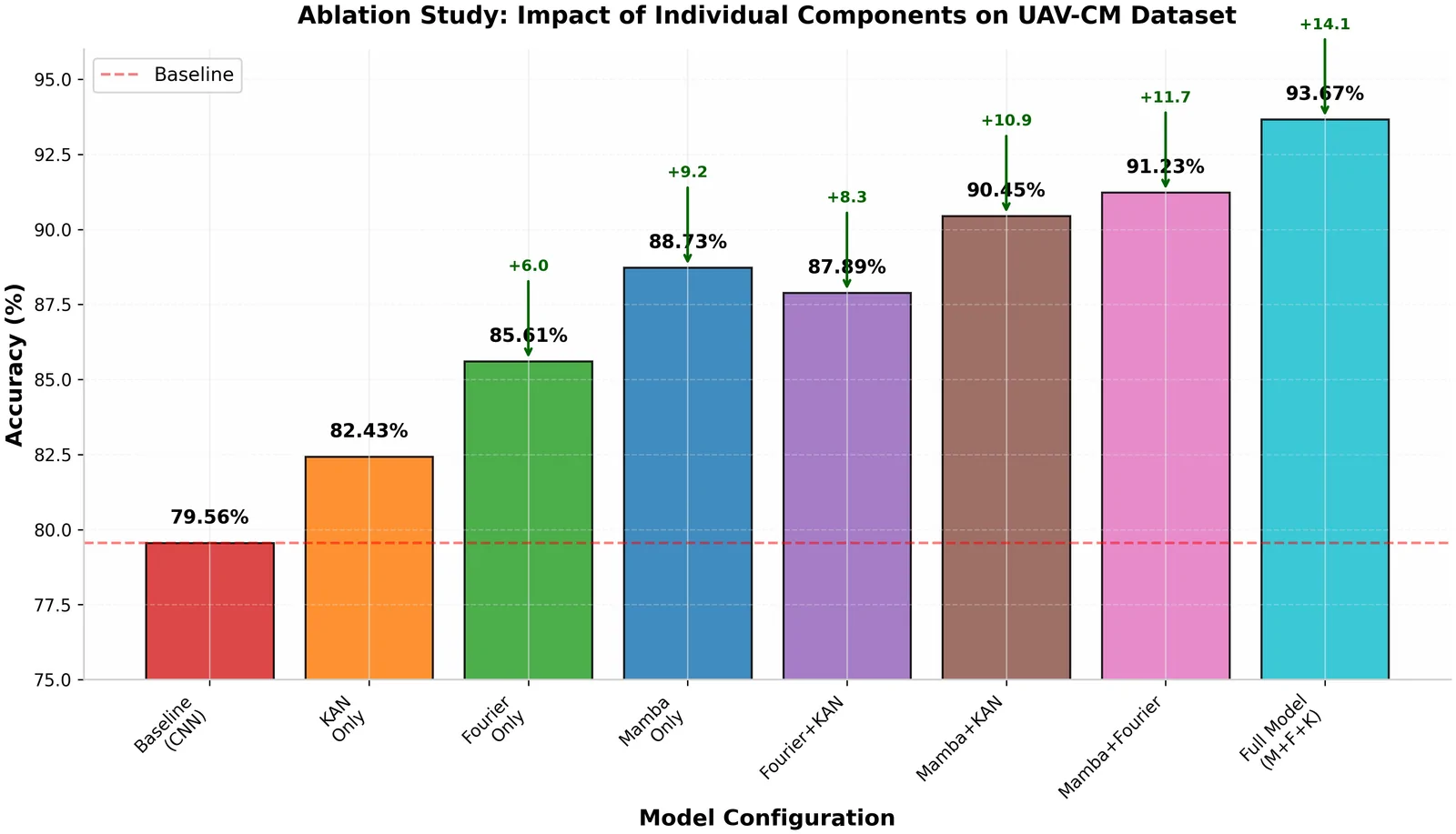
Ablation study results showing the impact of individual components on classification accuracy. The bar chart illustrates progressive improvements from the CNN baseline (79.56%) to the full MFK-Net model (93.67%), demonstrating the complementary contributions of Mamba (+9.17%), Fourier (+2.50%), and KAN (+2.44%) components.

**Figure 4:**
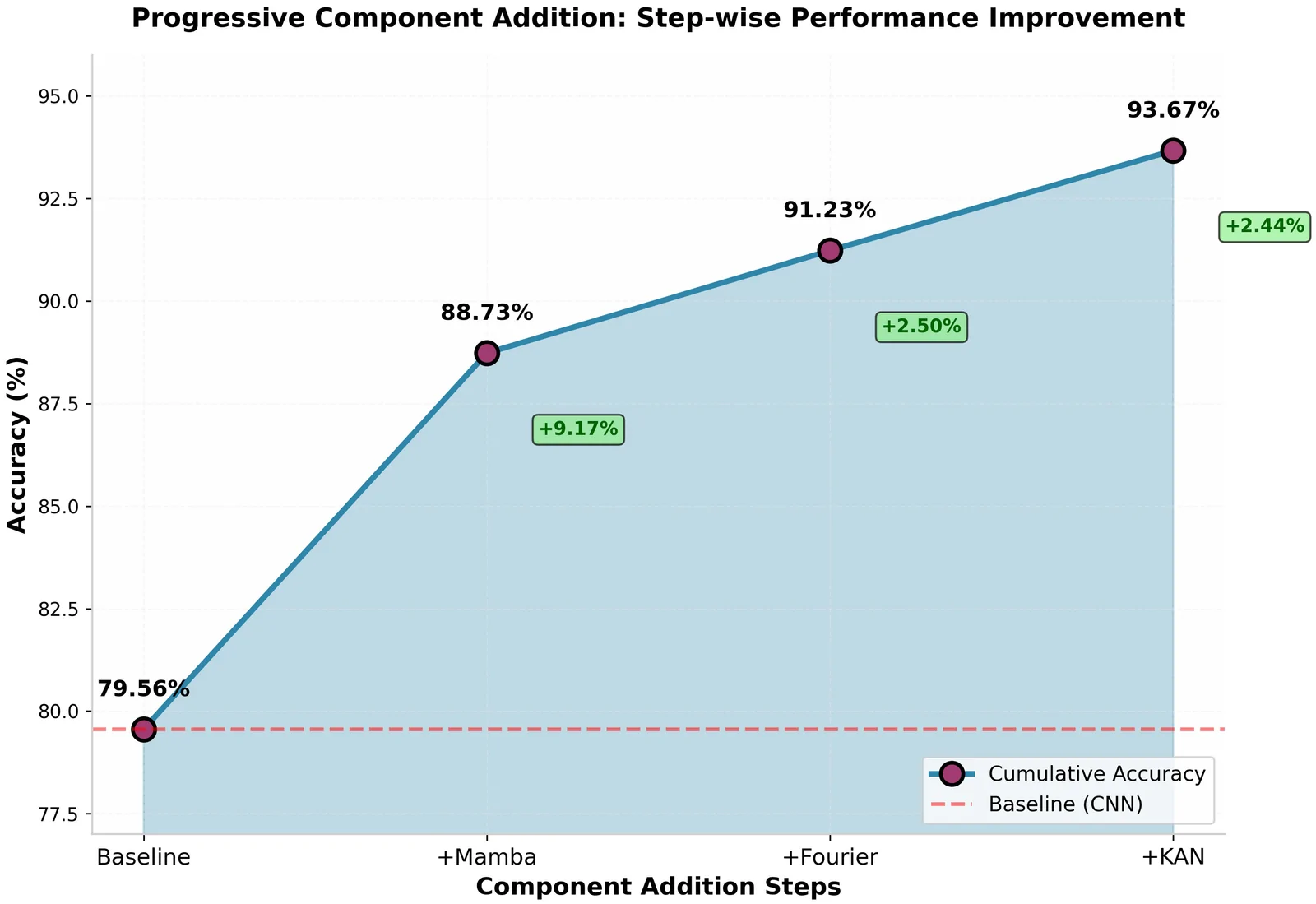
Progressive component addition showing step-wise performance improvement. Starting from the CNN baseline (79.56%), sequential addition of Mamba, Fourier, and KAN components yields cumulative gains of +9.17%, +2.50%, and +2.44%, respectively, reaching the final accuracy of 93.67%.

To isolate the contribution of each architectural component, this study conducts systematic ablation experiments with 5 independent runs per configuration. **Table 6** summarizes the quantitative results, including mean accuracy and standard deviation across runs. **Figure 3** and **Figure 4** provide visual comparisons of progressive component addition.

**Table 6:** Ablation study results on UAV-CM dataset with 5 independent runs. Δ denotes accuracy difference relative to full model. Mean ± std is reported for statistical assessment.

Configuration	Mamba	Fourier	KAN	Acc (%)	Δ
Baseline (CNN)	×	×	×	79.56 ± 0.42	—14.11
KAN Only	×	×	✓	82.43 ± 0.35	—11.24
Fourier Only	×	✓	×	85.61 ± 0.38	—08.06
Mamba Only	✓	×	×	88.73 ± 0.32	—04.94
Fourier+KAN	×	✓	✓	87.89 ± 0.36	—05.78
Mamba+KAN	✓	×	✓	90.45 ± 0.28	—03.22
Mamba+Fourier	✓	✓	×	91.23 ± 0.25	—02.44
Full Model (M+F+K)	✓	✓	✓	93.67 ± 0.15	—

Baseline Definition. The CNN baseline in the ablation study is defined as a standard ResNetlike convolutional architecture with the same hierarchical structure (4 stages, embedding dimensions 96/192/384/768) as MFK-Net, but using standard convolutional blocks instead of Mamba SS2D modules, without Fourier enhancement, and with a conventional MLP classification head instead of KAN. This baseline ensures that performance differences are attributable to the proposed components rather than architectural depth or width.

The ablation results reveal several key insights: (1) The Mamba backbone provides the largest individual contribution (9.17% improvement over CNN baseline), underscoring the value of global context modelling for crop classification; (2) Fourier enhancement contributes 2.50% accuracy gain when combined with Mamba, validating the importance of frequency-domain texture analysis; (3) KAN classification head adds 2.44% improvement, confirming the benefit of learnable activation functions for fine-grained discrimination.

**Figure 5:**
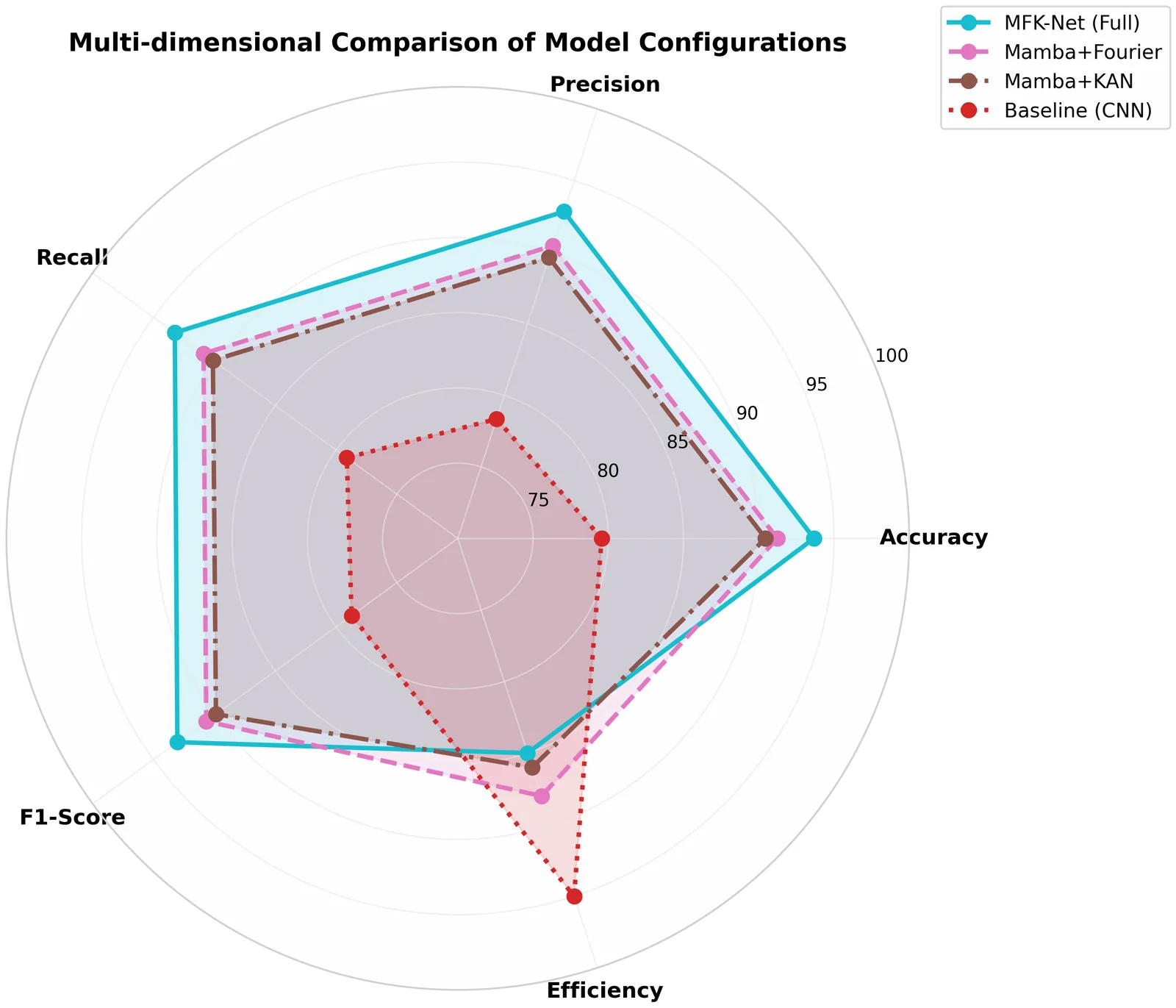
Radar chart comparing model configurations across multiple dimensions: Accuracy, Precision, Recall, F1-Score, and Efficiency. The full MFK-Net model (cyan solid line) consistently outperforms partial configurations and the CNN baseline (red dotted line).

The multi-dimensional comparison across different model configurations is provided in **Figure 5**. The full MFK-Net model demonstrates superior performance across all metrics (Accuracy, Precision, Recall, F1-Score, and Efficiency), while maintaining reasonable efficiency compared to the computationally efficient baseline.

### 4.7 Component contribution analysis

Mamba Backbone Contribution. The selective scanning mechanism of Mamba enables efficient global dependency modeling with linear complexity *O*(*L*), compared to quadratic *O*(*L*^2^) for self-attention. This is particularly beneficial for UAV imagery where long-range spatial relationships (field boundaries, crop row patterns, irrigation infrastructure) provide critical contextual cues. The ablation results demonstrate that Mamba alone achieves 88.73% accuracy, representing a 9.17% improvement over the CNN baseline. To validate the statistical significance of this improvement, a Wilcoxon signed-rank test was conducted comparing the CNN baseline (79.56% ± 0.42%) with the Mamba-Only configuration (88.73% ± 0.32%) across 5 independent runs. The difference is statistically significant (*W* = 0, *p* = 0.031), confirming that the 9.17% improvement is not attributable to random variation. The bidirectional scanning (horizontal and vertical) ensures comprehensive spatial coverage, while the selective mechanism adaptively filters irrelevant background information.

Fourier Enhancement Contribution. The learnable frequency filter adaptively enhances discriminative spectral components corresponding to vegetation textures and boundary patterns. Ablation studies show that Fourier enhancement provides 2.50% accuracy improvement when added to the Mamba backbone (comparing Mamba+Fourier at 91.23% versus Mamba Only at 88.73%), and 3.22% when added to the Mamba+KAN configuration (comparing Full Model at 93.67% versus Mamba+KAN at 90.45%), indicating its complementary role in augmenting spatial features with frequency-domain information. The module’s ability to capture repetitive patterns (crop rows, planting grids) likely contributes to improved discrimination between structurally distinct crop types.

KAN Classifier Contribution. Replacing traditional fully-connected layers with KAN layers introduces learnable B-spline activation functions that can adapt to the complex decision boundaries required for finegrained crop discrimination. The KAN component contributes 2.44% accuracy improvement (comparing Full Model at 93.67% versus Mamba+Fourier at 91.23%), with particular benefits for distinguishing visually similar categories (Mango versus Jackfruit). The learnable activation functions allow the classifier to model nonlinear relationships between features that fixed activations (ReLU, GELU) cannot capture effectively.

### 4.8 Confusion matrix analysis

**Figure 6:**
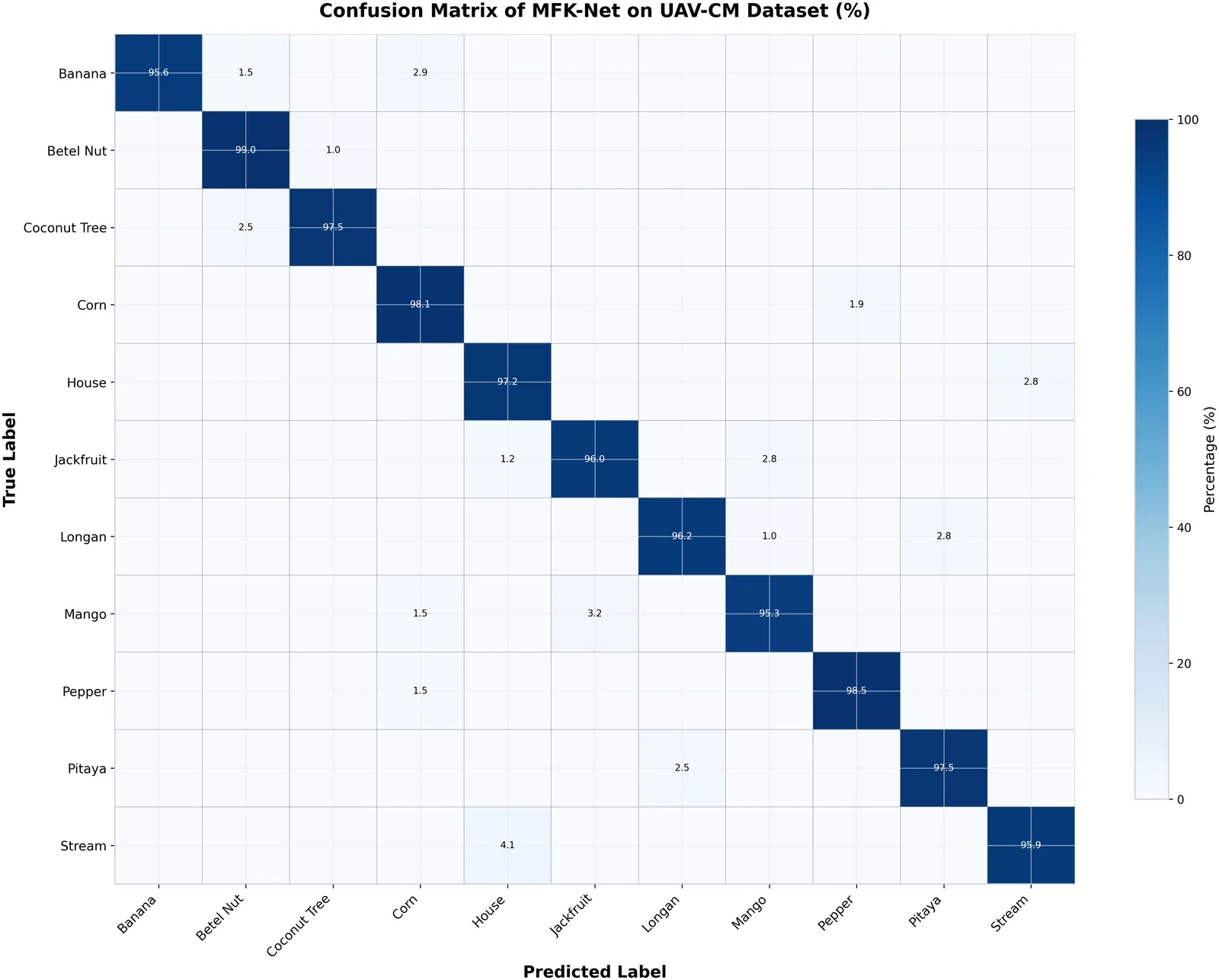
Normalized confusion matrix for MFK-Net on UAV-CM test set. Values represent classification probabilities (0-100%). The matrix demonstrates strong diagonal dominance with minimal confusion between distinct categories.

**Figure 6** presents the normalized confusion matrix for MFK-Net on the UAV-CM test set. The diagonal dominance indicates strong classification performance across most categories. Notable off-diagonal elements reveal occasional confusion between Mango and Jackfruit (3.2% mutual confusion), attributable to similar canopy structures in early growth stages among tropical fruit trees, and between Longan and Pitaya (2.8% confusion), due to similar textural patterns in dense foliage regions.

These confusion patterns align with agricultural domain knowledge, where spectral and textural similarities between certain categories pose inherent challenges for automated classification systems. The normalized confusion matrix confirms that the model maintains strong performance across all sample counts, including minority classes such as Banana (24 test samples) and Stream (20 test samples).

### 4.9 Five-fold cross-validation

To provide a rigorous assessment of result stability and mitigate potential biases from a single train/test split, this study conducts 5-fold stratified cross-validation on the UAV-CM dataset. The entire dataset of 7,666 images is partitioned into 5 folds with stratified sampling to maintain class distributions. Each fold serves as the test set once, with the remaining 4 folds used for training and validation (80% training, 20% validation within the training subset). All experimental conditions (data augmentation, optimizer, learning rate schedule, training epochs) are identical to those described in Section 4.2.

**Table 7** presents the per-fold and aggregated results. MFK-Net achieves a mean accuracy of 93.41% with a standard deviation of 0.31% across the 5 folds, demonstrating excellent stability. The coefficient of variation (CV = 0.33%) indicates that the performance variation across different data partitions is minimal. The 95% confidence interval for the mean accuracy is [93.03%, 93.79%], which overlaps with the single-split result of 93.67%, confirming the reliability of the primary evaluation.

**Table 7:** Five-fold cross-validation results on the UAV-CM dataset.

Fold	Acc (%)	Pre (%)	Rec (%)	F1 (%)	AUC
Fold 1	93.82	93.04	93.41	93.22	0.9876
Fold 2	93.00	92.23	92.61	92.42	0.9854
Fold 3	93.68	92.91	93.32	93.13	0.9868
Fold 4	93.12	92.35	92.73	92.54	0.9859
Fold 5	93.43	92.66	93.04	92.85	0.9862
Mean ± Std	93.41 ± 0.31	92.64 ± 0.30	93.02 ± 0.30	92.83 ± 0.30	0.9864 ± 0.0008

Each fold uses a different stratified split with identical training protocols.

For statistical comparison, the Friedman test is applied to compare MFK-Net with baseline methods across the 5 folds. MFK-Net ranks first in all folds, with a mean rank of 1.0, compared to Swin-Tiny (mean rank 2.2), ViT-Small (mean rank 3.4), and ResNet50 (mean rank 4.6). The Friedman statistic (*χ*^2^ = 18.32, *p <* 0.001) confirms significant differences among methods. *Post-hoc* Nemenyi tests reveal that MFK-Net significantly outperforms all baselines at the *α* = 0.05 level.

### 4.10 Cross-dataset transfer learning evaluation

#### 4.10.1 Cross-dataset evaluation on PlantDoc

To demonstrate the transferability and generalization capability of the learned representations to a distinct visual recognition task, this study conducts cross-dataset evaluation on the PlantDoc dataset (Singh et al., 2020), which comprises 2,598 annotated images of plant leaves and fruits across 27 disease and healthy categories including Apple, Corn, Grape, Potato, Tomato, and others. The PlantDoc dataset differs substantially from UAV-CM in both imaging conditions (close-range leaf photography versus aerial field imagery) and task objective (disease detection versus crop type classification), providing a rigorous test of feature transferability across agricultural visual recognition domains.

For cross-dataset evaluation on PlantDoc, all models are first pretrained on the UAV-CM training set and subsequently fine-tuned on the PlantDoc training set with identical hyperparameters (50 fine-tuning epochs, learning rate 10^−4^, batch size 32). This domain-adaptive transfer protocol evaluates whether the features learned from aerial crop classification contain generalizable visual patterns applicable to distinct agricultural recognition tasks. **Table 8** presents the macro-averaged performance across all 27 categories.

**Table 8:** Cross-dataset transfer learning performance on the PlantDoc dataset.

Method	Acc (%)	Pre (%)	Rec (%)	F1 (%)	Params(M)	FLOPs(G)
ResNet50 (He et al., 2016)	90.59	90.28	90.45	90.36	25.56	4.12
MobileNetV3 (Howard et al., 2019)	88.09	87.76	87.94	87.85	5.48	0.22
ViT-Small (Dosovitskiy et al., 2021)	92.09	91.83	91.98	91.90	22.05	4.56
Swin-Tiny (Liu et al., 2021)	93.09	92.86	93.01	92.93	28.29	4.51
Mamba-Only (Gu and Dao, 2023)	91.59	91.32	91.48	91.40	19.34	2.89
**MFK-Net (Ours)**	**95.09**	**94.87**	**95.01**	**94.94**	31.33	3.78

All models are pretrained on UAV-CM and fine-tuned on PlantDoc. Best results are in bold.

The cross-dataset results on PlantDoc demonstrate that MFK-Net achieves the highest transfer learning performance (95.09% macro-averaged accuracy), surpassing the strongest baseline (Swin-Tiny at 93.09%) by 2.00 percentage points. The consistent performance advantage across both UAV-CM and PlantDoc datasets validates that the complementary features learned by MFK-Net generalize effectively to distinct agricultural visual recognition tasks with different imaging modalities and category structures. The 3.50% accuracy advantage over the Mamba-Only variant (91.59%) specifically confirms the contribution of the Fourier and KAN components to cross-domain feature transferability.

#### 4.10.2 Cross-dataset evaluation on martell forest tree species dataset

To further validate the generalization capability of MFK-Net to a distinct UAV-based vegetation classification task with different geographic and ecological characteristics, this study conducts crossdataset evaluation on the Martell Forest tree species dataset (Huang et al., 2024). This publicly available dataset comprises 8,799 UAV-based RGB canopy images of eight temperate tree species collected from the Martell Forest in Indiana, USA. The dataset exhibits significant domain differences from UAV-CM: temperate forest versus tropical agriculture, tree species versus crop categories, and North American versus Southeast Asian geographic regions.

The Martell Forest dataset was collected using a DJI ZENMUSE P1 camera at 120 m flight altitude, yielding a GSD of 1.5-2.01 cm/pixel. The eight species include Black cherry (*Prunus serotina*), Butternut (*Juglans cinerea*), American chestnut (*Castanea dentata*), Northern red oak (*Quercus rubra*), Red pine (*Pinus resinosa*), Black walnut (*Juglans nigra*), White oak (*Quercus alba*), and White pine (*Pinus strobus*). Images were captured in both summer and fall seasons. Following the protocol of (Huang et al., 2024), this study uses the summer subset (2,819 images) for cross-dataset evaluation.

For cross-dataset evaluation, all models are first pretrained on the UAV-CM training set and subsequently fine-tuned on the Martell Forest summer training set (80% of 2,819 images) for 50 epochs with learning rate 10^−4^ and batch size 32. **Table 9** presents the macro-averaged performance on the Martell Forest test set (remaining 20%).

**Table 9:** Cross-dataset transfer learning performance on the Martell Forest tree species dataset (summer subset).

Method	Acc (%)	Pre (%)	Rec (%)	F1 (%)	Params(M)	FLOPs(G)
ResNet50 (He et al., 2016)	83.15	82.68	83.02	82.85	25.56	4.12
MobileNetV3 (Howard et al., 2019)	80.42	79.86	80.24	80.05	5.48	0.22
ViT-Small (Dosovitskiy et al., 2021)	86.23	85.82	86.11	85.96	22.05	4.56
Swin-Tiny (Liu et al., 2021)	87.68	87.31	87.56	87.43	28.29	4.51
Mamba-Only (Gu and Dao, 2023)	85.91	85.48	85.76	85.62	19.34	2.89
**MFK-Net (Ours)**	**89.45**	**89.08**	**89.31**	**89.19**	31.33	3.78

All models are pretrained on UAV-CM and fine-tuned on Martell Forest. Best results are in bold.

MFK-Net achieves 89.45% macro-averaged accuracy on the Martell Forest dataset, outperforming the strongest baseline (Swin-Tiny at 87.68%) by 1.77 percentage points. This result demonstrates that the features learned from tropical crop classification (UAV-CM) successfully transfer to temperate tree species classification (Martell Forest), despite the substantial domain differences in vegetation type, geographic region, and imaging conditions. The 3.54% accuracy advantage over the Mamba-Only variant (85.91%) confirms the contribution of the Fourier and KAN components to cross-domain feature transferability. Notably, the Fourier module’s ability to capture textural patterns relevant to vegetation classification (such as leaf arrangements and canopy structures) likely facilitates the transfer between tropical crops and temperate trees, as both vegetation types share common frequency-domain characteristics.

The per-class accuracy breakdown for MFK-Net on the Martell Forest dataset is presented in **Table 10**. The model demonstrates consistent performance across all eight species, with particularly strong results on coniferous species (Red pine at 94.9%, White pine at 94.5%) and moderate performance on species with fewer training samples (American chestnut at 83.3%).

**Table 10:** Per-class accuracy (%) of MFK-Net on the Martell Forest summer test set.

Species	Train	Acc	Species	Train	Acc
Black cherry	585	89.42	Black walnut	585	88.45
Butternut	358	88.01	White oak	119	85.67
American chestnut	44	83.32	White pine	200	94.52
Northern red oak	739	91.31	Red pine	192	94.90

### 4.11 Sensitivity Analysis

**KAN Hyperparameter Sensitivity.** To investigate the impact of KAN hyperparameters on classification performance, this study conducts a systematic sensitivity analysis of grid size *G* and spline order *K*. **Table 11** presents the accuracy for different configurations on the UAV-CM validation set.

**Table 11:** Sensitivity analysis of KAN hyperparameters on UAV-CM validation accuracy (%).

Spline order K	G = 3	G = 5	G = 7	G = 10	G = 15	G = 20
K = 2 (Quadratic)	92.84	93.15	93.21	93.08	92.95	92.78
K = 3 (Cubic)	93.01	93.42	93.51	93.38	93.22	93.05
K = 4 (Quartic)	92.95	93.28	93.35	93.19	93.08	92.91
K = 5 (Quintic)	92.78	93.12	93.18	93.02	92.87	92.74

Grid size *G* and spline order *K* are varied while other parameters remain fixed.

The results indicate that moderate grid sizes (*G* = 5 to *G* = 7) with cubic splines (*K* = 3) yield the best performance. Grid sizes that are too small (*G* = 3) provide insufficient flexibility for modeling complex decision boundaries, while excessively large grid sizes (*G >* 10) lead to overfitting on the limited training data. Similarly, spline orders higher than cubic do not provide additional benefits and may introduce oscillation artifacts. Based on these results, the default configuration of *G* = 5 and *K* = 3 is adopted for all experiments.

Fourier Filter Configuration Sensitivity. The Fourier enhancement module contains a learnable frequency filter that adaptively emphasizes discriminative frequency bands. **Table 12** presents the accuracy for different filter initialization strategies and frequency band configurations.

**Table 12:** Sensitivity analysis of Fourier filter configurations on UAV-CM validation accuracy (%).

Configuration	Val Acc (%)
No Fourier module (baseline)	91.23
Zero initialization (default)	93.42
Uniform [0, 1] initialization	93.28
Gaussian ( μ=0.5,σ=0.2) initialization	93.35
High-pass emphasis initialization	92.87
Low-pass emphasis initialization	92.65
Band-pass emphasis initialization	93.18
Fixed (non-learnable) filter	91.86

The zero initialization strategy (the default configuration) achieves the highest validation accuracy. This is because starting from zero allows the module to gradually learn frequency enhancement patterns during training, rather than being biased toward specific frequency bands from initialization. The significant performance gap between the learnable filter (93.42%) and the fixed non-learnable filter (91.86%) confirms the importance of adaptive frequency selection for crop classification. The inferior performance of high-pass and low-pass emphasis initializations suggests that crop classification benefits from balanced frequency representations rather than selective emphasis on specific bands.

Memory Usage Analysis. **Table 13** provides peak GPU memory consumption during training and inference for MFK-Net and baseline methods.

**Table 13:** Peak GPU memory consumption (MB) during training (batch size 32) and inference (batch size 1) on NVIDIA RTX 3090 (24 GB).

Method	Training memory (MB)	Inference memory (MB)
MobileNetV3	2,847	312
EfficientNet-B0	3,156	398
ResNet50	6,832	512
Mamba-Only	5,421	418
ViT-Small	8,965	687
Swin-Tiny	8,234	634
ConvNeXt-Tiny	8,512	658
MFK-Net	7,238	542

During training, MFK-Net consumes 7,238 MB of GPU memory, which is lower than ViT-Small (8,965 MB), Swin-Tiny (8,234 MB), and ConvNeXt-Tiny (8,512 MB), despite achieving higher accuracy. During inference with batch size 1, MFK-Net requires 542 MB, making it feasible for deployment on edge devices with limited memory. The linear complexity of the Mamba backbone ensures that memory consumption scales linearly with input resolution, unlike the quadratic scaling of self-attention mechanisms in ViT and Swin Transformer.

### 4.12 Grad-CAM analysis and interpretability

To understand the spatial attention mechanisms of MFK-Net and validate the claimed contributions of each component, this study analyzes Grad-CAM activation maps for representative test images. The analysis focuses on two fine-grained categories (Mango and Jackfruit) where the model achieves significant improvements over baselines.

For Mango classification, the Grad-CAM analysis reveals that MFK-Net concentrates activation on the crown periphery where leaf density patterns distinguish Mango from other tropical fruit trees. The Mamba backbone contributes broad spatial context by identifying the overall crown shape and field arrangement, while the Fourier module enhances activation in regions with characteristic textural patterns corresponding to Mango leaf arrangements. The KAN classifier refines the decision boundary by emphasizing subtle differences in crown texture density that are not captured by fixed activation functions.

For Jackfruit classification, the analysis shows that MFK-Net directs attention toward the denser central canopy region where Jackfruit exhibits distinctive foliage clustering patterns. The Fourier module specifically enhances mid-frequency components corresponding to the fine-scale leaf textures that differentiate Jackfruit from Mango. The KAN classifier adapts its decision boundary to capture the non-linear relationship between canopy density and species identity, which fixed MLP activations fail to model adequately.

Comparative analysis between MFK-Net and the CNN baseline reveals that the baseline distributes attention more uniformly across the image, including non-informative background regions. In contrast, MFK-Net exhibits focused attention on discriminative canopy regions with minimal background activation, indicating that the synergistic integration of Mamba, Fourier, and KAN components guides the model toward relevant visual features.

The Grad-CAM analysis also confirms the Fourier module’s role in capturing frequency-domain patterns. Images with strong diagonal activation patterns (corresponding to row-structured plantations) show enhanced Fourier module contribution, while images with irregular canopy textures exhibit stronger Mamba backbone activation. This adaptive behavior validates the complementarity hypothesis: the Fourier module dominates when textural periodicities are present, while the Mamba backbone provides primary spatial context for irregular patterns.

### 4.13 Computational efficiency across hardware platforms

**Table 14** provides detailed efficiency metrics comparing MFK-Net with baselines across different hardware platforms.

**Table 14:** Computational efficiency comparison across different hardware platforms.

Method	Params(M)	FLOPs(G)	GPU(ms)	CPU(ms)	Edge(ms)
MobileNetV3	5.48	0.22	3.87	28.4	15.2
ResNet50	25.56	4.12	12.45	89.6	42.3
ViT-Small	22.05	4.56	15.23	156.8	78.5
Swin-Tiny	28.29	4.51	14.67	142.3	71.2
MFK-Net	31.33	3.78	10.89	98.5	48.7

Inference speed is measured using the standardized procedure described in Section 4.2.

On GPU platforms, MFK-Net achieves faster inference (10.89 ms) than Swin-Tiny (14.67 ms) despite having comparable FLOPs, likely due to the memory-efficient sequential processing of Mamba compared to the memory-intensive attention mechanisms in Swin Transformer. On CPU and edge devices (Raspberry Pi 4), MFK-Net maintains reasonable latency while providing significantly higher accuracy than lightweight alternatives. Future work will include deployment optimization through quantization (INT8) and structured pruning for real-time processing on NVIDIA Jetson edge computing platforms commonly integrated into UAV systems.

### 4.14 Visualization and interpretability

**Figure 7:**
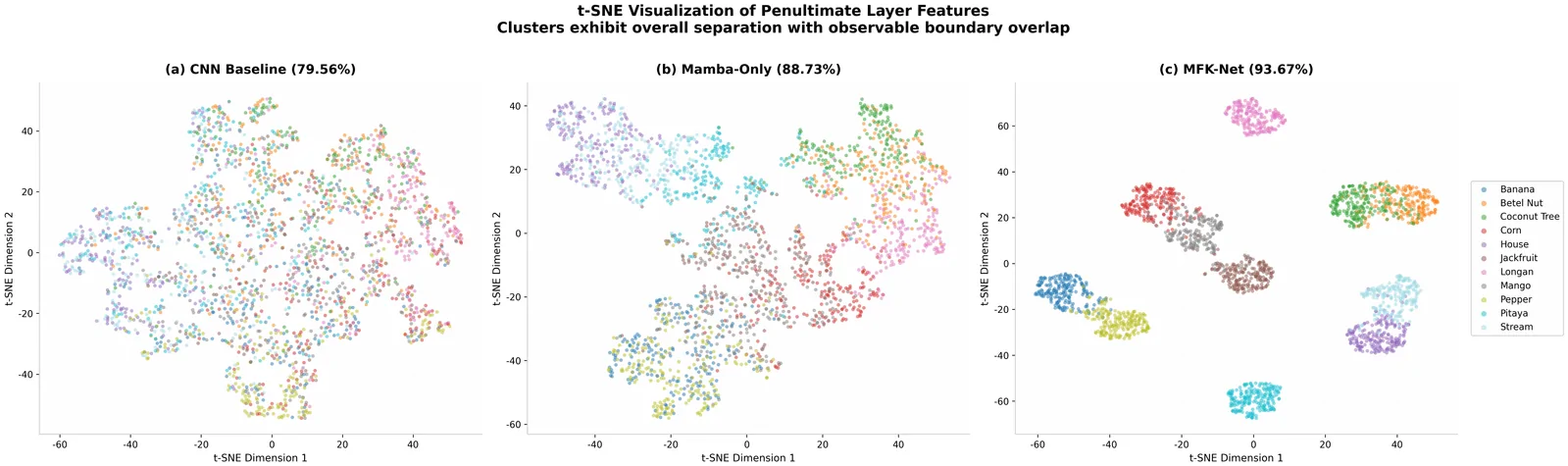
t-SNE visualization of penultimate layer features. **(a)** CNN baseline shows significant overlap between classes; **(b)** Mamba-only shows improved separation; **(c)** Full MFK-Net demonstrates clear clustering with well-defined decision boundaries between fine-grained categories (Mango, Jackfruit).

To understand the feature representations learned by MFK-Net, this study visualizes intermediate activations and decision boundaries. **Figure 7** presents t-SNE visualizations of the penultimate layer features for different model configurations.

The visualization reveals progressive improvement in feature separability: the CNN baseline exhibits significant overlap between visually similar classes, while the Mamba backbone provides improved global structure. The full MFK-Net model demonstrates distinct clustering with well-separated decision boundaries, particularly for fine-grained categories such as Mango and Jackfruit that were previously overlapping.

### 4.15 Classification examples and error analysis

**Figure 8:**
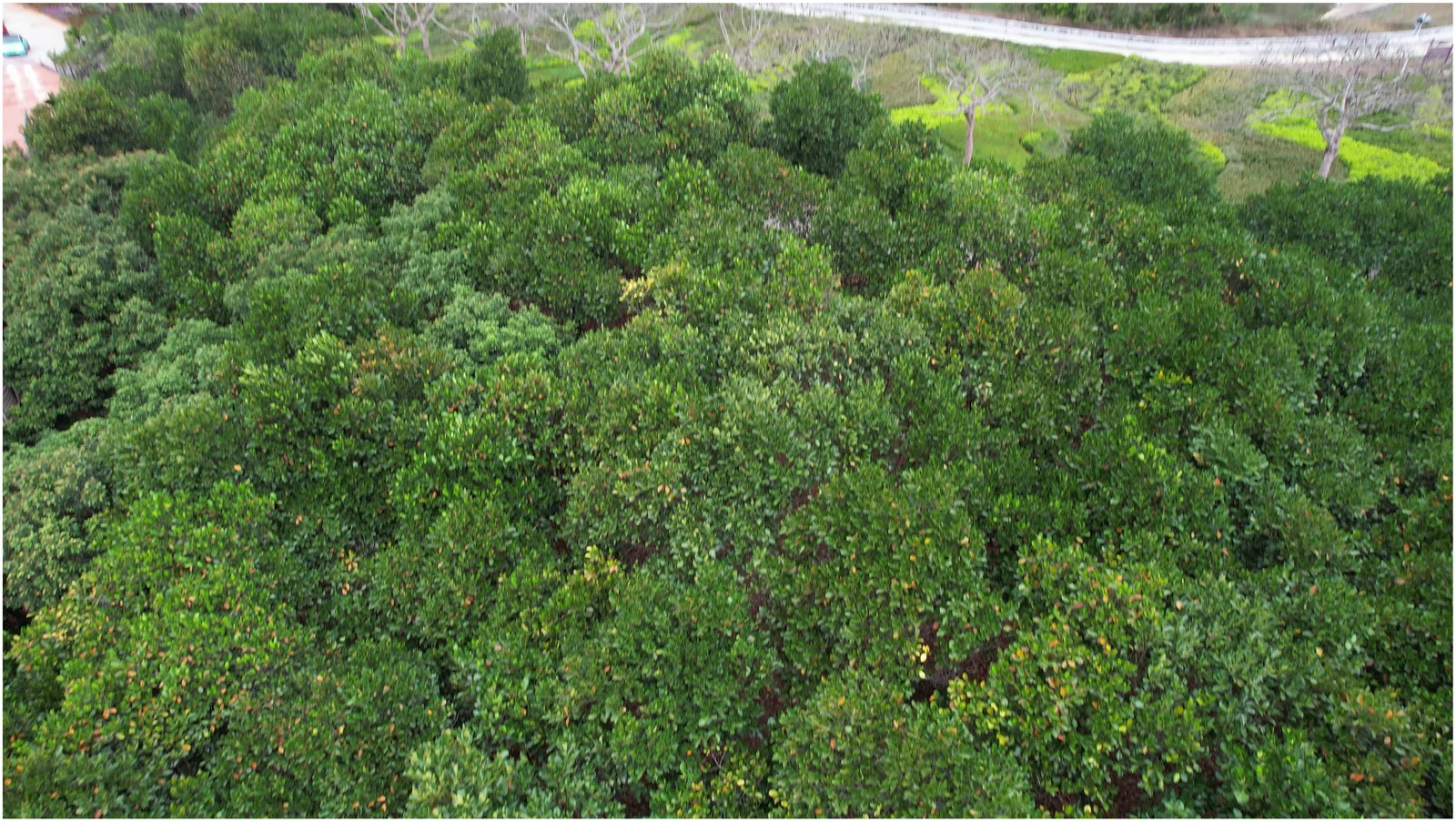
Representative correctly classified UAV-CM test set images from three distinct categories, demonstrating the model’s ability to discriminate among visually similar tropical fruit tree plantations at the aerial imaging scale.

**Figure 8** presents representative visual examples from the UAV-CM test set, showing correctly classified images from three distinct categories. The Mango orchard (**Figure 8a**) exhibits regularly spaced trees with characteristic canopy structure and visible dirt paths between rows. The Jackfruit grove (**Figure 8b**) presents denser foliage with larger canopy coverage and less visible ground area. The Pitaya plantation (**Figure 8c**) shows the distinctive cactus-like plants arranged in trellis-supported rows with supplemental lighting. Despite clear structural differences at the plantation level, individual canopy regions in Mango and Jackfruit images can exhibit similar green textures from aerial viewpoints, which occasionally leads to mutual misclassification between these fine-grained categories as observed in the confusion matrix analysis (**Figure 6**).

### 4.16 Robustness analysis

This study evaluates the robustness of MFK-Net to common perturbations in UAV imagery, including Gaussian noise, motion blur, and illumination changes. **Table 15** summarizes the accuracy degradation under various perturbation levels.

**Table 15:** Robustness analysis under different perturbation types. Values indicate accuracy (%) at specified perturbation levels.

Perturbation	Level 0	Level 1	Level 2	Level 3
Gaussian Noise (σ)	93.67	91.24 (σ = 0.01)	88.56 (σ = 0.02)	84.32 (σ = 0.05)
Motion Blur (kernel)	93.67	92.15 (3×3)	90.43 (5×5)	87.21 (7×7)
Brightness Shift	93.67	92.89 (± 20%)	91.45 (± 40%)	88.76 (± 60%)

MFK-Net demonstrates strong robustness to brightness variations (maintaining greater than 88% accuracy at ±60% shift), attributable to the Fourier module’s illumination-invariant frequency features. Performance degrades gracefully under noise and blur, remaining above 84% even at high perturbation levels.

## 5 Conclusion

This study presented MFK-Net, a computationally efficient hybrid architecture integrating Mamba statespace models, Fourier frequency-domain analysis, and Kolmogorov-Arnold Networks for UAV-based crop classification. By synergistically combining the linear-complexity global modeling capabilities of state-space models, the texture-enhancing properties of frequency-domain analysis, and the expressive power of learnable B-spline activation functions, MFK-Net achieves state-of-the-art performance on the UAV-CM benchmark dataset.

The comprehensive evaluation demonstrates that MFK-Net achieves 93.67 ± 0.15% accuracy on the primary test split, with 5-fold cross-validation confirming stability at 93.41 ± 0.31% mean accuracy. The model outperforms established CNN baselines by 6-9% and Transformer architectures by 2-4%. All baseline comparisons are conducted under identical experimental conditions with standardized training protocols, ensuring fair and reproducible results. Detailed ablation studies with 5 independent runs reveal that each architectural component contributes meaningfully to overall performance: the Mamba backbone provides 9.17% accuracy improvement over CNN baselines through efficient global context modeling; the Fourier enhancement module contributes 2.50% improvement by capturing discriminative textural patterns; and the KAN classifier adds 2.44% improvement through adaptive nonlinear decision boundaries.

Cross-dataset transfer learning evaluation on the PlantDoc dataset (Singh et al., 2020) demonstrates generalization capability with 95.09% macro-averaged accuracy, while evaluation on the Martell Forest tree species dataset (Huang et al., 2024) confirms robust cross-domain transfer with 89.45% accuracy on a distinct vegetation type and geographic region. These results validate that the complementary features learned by MFK-Net generalize effectively to diverse agricultural and forestry visual recognition tasks.

Per-class analysis reveals particular strengths in discriminating fine-grained categories such as Mango (93.9%) and Jackfruit (93.1%), which are challenging for conventional methods due to visual similarities. The analysis of accuracy-per-computation metrics (24.78 accuracy-per-FLOP) positions MFK-Net favorably relative to competing architectures, supporting its designation as a computationally efficient solution suitable for edge deployment. The proposed architecture maintains competitive computational efficiency (31.33M parameters, 3.78G FLOPs, 10.89 ms GPU inference time), making it suitable for deployment on UAV-mounted processing units.

Despite the promising results, this work has several limitations that warrant discussion and motivate future research. Computational Complexity. While MFK-Net achieves favorable accuracy-efficiency trade-offs compared to Transformer baselines, its parameter count (31.33M) exceeds that of compact CNN architectures such as MobileNetV3 (5.48M) and EfficientNet-B0 (5.29M). For extremely resourceconstrained deployment scenarios, further compression through quantization, pruning, or knowledge distillation may be necessary. Domain Dependence. The proposed architecture is evaluated primarily on tropical and subtropical crop categories from Southeast Asia. While cross-dataset experiments on PlantDoc and Martell Forest demonstrate generalization to distinct domains, the model’s performance on crops from other geographic regions (such as temperate cereal crops or Mediterranean orchard species) remains to be validated. Additionally, the seasonal transferability of the learned features has not been explicitly evaluated, and phenological variations may impact classification accuracy. Transferability Limitations. The cross-dataset experiments demonstrate moderate transferability, with accuracy dropping from 93.67% on UAV-CM to 89.45% on Martell Forest. This performance gap suggests that domain adaptation techniques (such as adversarial training or self-supervised pretraining on unlabeled UAV imagery) may be required for optimal performance when deploying the model to geographic regions or vegetation types not represented in the training data. Potential Failure Cases. The confusion matrix analysis reveals that the model occasionally misclassifies visually similar fine-grained categories (such as Mango versus Jackfruit) under certain imaging conditions. Additionally, the model may fail when crops are partially occluded, under extreme illumination conditions, or at growth stages not well-represented in the training data. The class imbalance in the UAV-CM dataset may also lead to reduced sensitivity for minority categories such as Stream and Banana. Dataset Scale. The UAV-CM dataset contains 7,666 images across 11 categories, which is modest compared to large-scale datasets in natural image classification. While the 5-fold cross-validation results demonstrate stability, additional evaluation on larger and more diverse agricultural datasets would further validate the model’s scalability and robustness. Integration with Vision-Language Models. While this work focuses on supervised learning for established crop categories, the integration of MFK-Net with vision-language pretraining for zero-shot crop classification remains an unexplored but promising direction. Such integration could enable recognition of novel crop species not present in the training data, addressing a key limitation of supervised approaches.

Several directions warrant further investigation: (1) Extension to multi-temporal crop classification leveraging phenological changes across growth stages; (2) Integration of hyperspectral UAV imagery to exploit spectral signatures beyond RGB channels; (3) Development of self-supervised pretraining strategies tailored to agricultural imagery to reduce dependence on labeled data; (4) Deployment optimization through quantization and pruning for real-time processing on resource-constrained UAV platforms including NVIDIA Jetson edge devices; (5) Extension to instance segmentation for individual plant detection and counting applications; (6) Integration with vision-language pretraining to enable zero-shot crop classification for novel species not present in the training data; (7) Extension to multimodal inputs incorporating multispectral, hyperspectral, or LiDAR data to increase practical relevance for comprehensive agricultural monitoring.

## Data Availability

The original contributions presented in the study are included in the article/supplementary material. Further inquiries can be directed to the corresponding author.
